# Regio- and stereoselective synthesis of benzothiazolo-pyrimidinones *via* an NHC-catalyzed Mannich/lactamization domino reaction†
†Electronic supplementary information (ESI) available. CCDC 1029497. For ESI and crystallographic data in CIF or other electronic format see DOI: 10.1039/c4cc08594a



**DOI:** 10.1039/c4cc08594a

**Published:** 2014-12-05

**Authors:** Qijian Ni, Xiaoxiao Song, Jiawen Xiong, Gerhard Raabe, Dieter Enders

**Affiliations:** a Institute of Organic Chemistry , RWTH Aachen University , Landoltweg 1 , 52074 Aachen , Germany . Email: enders@rwth-aachen.de

## Abstract

An NHC-catalyzed regio- and stereoselective Mannich/lactamization domino reaction of *N*-(benzothiazolyl)imines with α-chloroaldehydes has been developed. This new protocol provides a facile approach for the asymmetric synthesis of benzothiazolo-pyrimidinones and a pyrrolo[1,2*-a*]indolone in moderate to good yields (34–78%) and excellent stereoselectivities (87–99% ee, up to >20 : 1 d.r.).

## Abstract 2

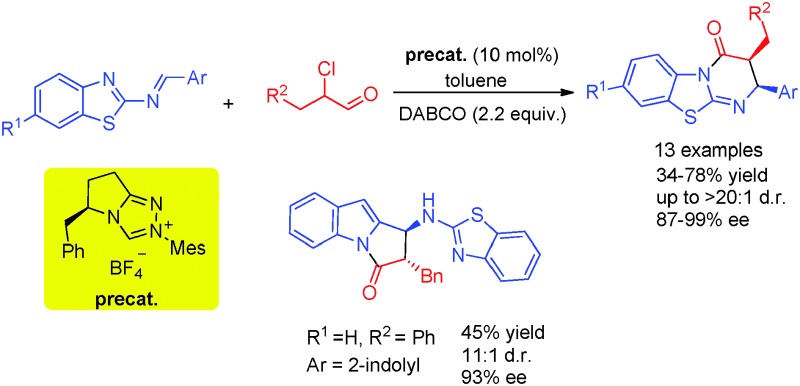
An NHC-catalyzed regio- and stereoselective Mannich/lactamization domino reaction of *N*-(benzothiazolyl)imines with α-chloroaldehydes has been developed.

The tricyclic pyrimido[2,1-*b*]benzothiazole core prevails in a wide range of bioactive molecules with remarkable biological properties,^1^ such as the inhibition of *c*-AMP phosphodiesterase, antineoplastic and antimalarial activity. Moreover, the isothiourea-based HBTM is used as an efficient organocatalyst and received great attention in the field of asymmetric catalysis (Fig. 1).^2^ Although various approaches for the synthesis of the pyrimido[2,1-*b*]benzothiazole motif have been developed, most of them are non-stereoselective and/or need relatively harsh conditions.^3^

**Fig. 1:**
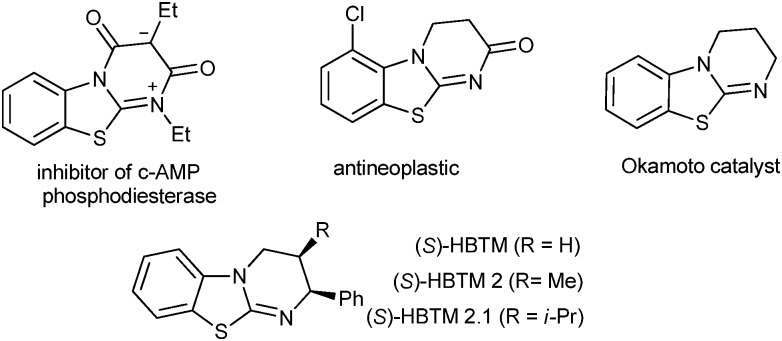
Examples of pyrimido[2,1-*b*]benzothiazole derivatives.

In the past decade, great advances have been achieved in the development of N-heterocyclic carbene (NHC) catalyzed organocatalytic reactions *via* the umpolung of aldehydes.^4^ Especially since the seminal studies concerning the NHC-catalyzed conjugate umpolung reactions reported by the groups of Glorius and Bode in 2004,^5^ NHC organocatalysis has been extended for the activation of the β-carbon (homoenolate intermediate) and α-carbon (enolate intermediate) of enals. These two kinds of intermediates used as nucleophiles reacted with a variety of reactive electrophiles to afford heterocyclic compounds such as lactones, lactams or cyclopentenes.^6^ It is noteworthy that aldimines behaved as excellent electrophiles in the reactions of enolate intermediates, providing the corresponding β-lactams (Scheme 1, eqn (1)). Smith *et al.* ^7^ and Ye *et al.* ^8^ reported an NHC-catalyzed [2+2] cycloaddition of ketenes with *N*-tosyl imines and *N*-Boc imines, respectively. Very recently, the Ye group was able to carry out Staudinger reactions of ketenes with isatin-derived ketimines, yielding spirocyclic oxindolo-β-lactams.^9^ We envisioned that 2-benzothiazolimine **1a** in combination with an azolium enolate could not only produce the β-lactam **4**, but also provide access to benzothiazolo-pyrimidinone **3** through a formal [4+2] annulation (Scheme 1, eqn (2)). Obviously, influencing the regioselectivity of the reaction site of the intermediate ambident anions is the greatest challenge in order to improve the ratio of **3**/**4** in the Mannich/lactamization domino reaction.

**Scheme 1:**
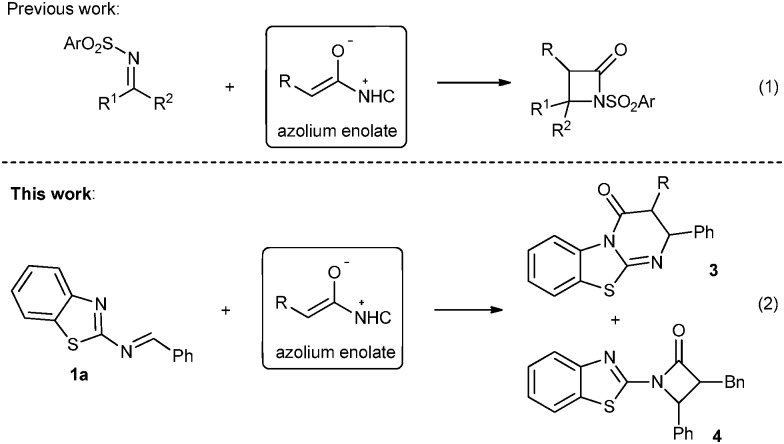
Reactions of imines with NHC-bound enolate intermediates.

To test our hypothesis, we initially checked several triazolium precatalysts **A–C** in the model reaction of 2-benzothiazolimine **1a** with 2-chloro-3-phenylpropanal (**2a**) at room temperature. We found that the chiral triazolium salt **B** resulted in an excellent stereoselectivity for *ent*-**3a** (97% ee, >20 : 1 d.r.), albeit with a low yield (19%) and regioselectivity (Table 1, entry 2). Attempting to improve the regioselectivity and the yield of *ent*-**3a**, we next tested a series of bases, but no satisfying improvement was achieved (Table 1, entries 4–9). Solvent screening led to no improvement in yield and selectivity (Table 1, entries 10–14). After the screening of the base and solvent, DABCO in combination with toluene turned out to give the highest yield of *ent*-**3a** (31%), maintaining the excellent stereoselectivity and the low regioselectivity as well (Table 1, entry 6). Notably, the triazolium salt **A** provided *rac*-**3a** in 43% yield exclusively, even though with a drastically decreased d.r. (Table 1, entry 1). Therefore we further screened a series of pyrrolidinone-derived triazolium salts **D–G**. To our delight, a dramatic improvement in both yield and regioselectivity was obtained (Table 1, entries 15–18). The NHC-catalyzed reaction based on the triazolium salt **F** afforded cycloadduct **3a** in a better yield but with relatively low regioselectivity (Table 1, entry 17). After using 4 Å MS as an additive, the yield (69%) and regioselectivity (14 : 1) improved further, but the enantioselectivity (93% ee) decreased slightly (Table 1, entry 19).

**Table 1:** Optimization of the reaction conditions^*a*^

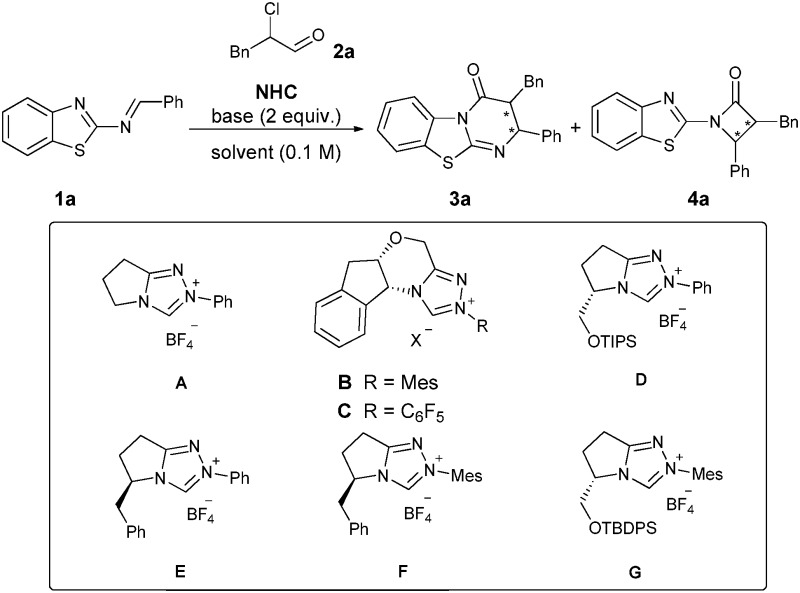							
Entry	NHC	Base	Solvent	Yield of **3a** ^*b*^ (%)	**3a**/**4a** ^*c*^	d.r. (**3a**)^*d*^	ee of **3a** ^*e*^ (%)
1	**A**	NEt_3_	Toluene	43	>20 : 1	1.3 : 1	—
2	**B**	NEt_3_	Toluene	19	1 : 1.6	>20 : 1	–97
3	**C**	NEt_3_	Toluene	—	—	—	—
4	**B**	DIPEA	Toluene	30	1.4 : 1	>20 : 1	–99
5	**B**	TMEDA	Toluene	23	1 : 1.7	>20 : 1	–98
6	**B**	DABCO	Toluene	31	1 : 1.6	>20 : 1	–94
7	**B**	DBU	Toluene	—	—	—	—
8	**B**	K_2_CO_3_	Toluene	15	1 : 1.2	>20 : 1	–97
9	**B**	NaOAc	Toluene	—	—	—	—
10	**B**	DABCO	THF	24	1 : 1.3	>20 : 1	–99
11	**B**	DABCO	DCM	22	1 : 1.4	>20 : 1	–98
12	**B**	DABCO	EtOAc	30	1 : 1.2	>20 : 1	–99
13	**B**	DABCO	PhCl	14	1 : 1.7	>20 : 1	–98
14	**B**	DABCO	Dioxane	22	1 : 1.9	>20 : 1	–97
15	**D**	DABCO	Toluene	27	>20 : 1	>20 : 1	–90
16	**E**	DABCO	Toluene	58	>20 : 1	>20 : 1	90
17	**F**	DABCO	Toluene	65	7.2 : 1	>20 : 1	97
18	**G**	DABCO	Toluene	54	>20 : 1	>20 : 1	–92
19^*f*^	**F**	DABCO	Toluene	69	14 : 1	>20 : 1	93

^*a*^Reaction conditions: **1a** (0.1 mmol), **2a** (0.2 mmol), NHC (0.01 mmol), base (0.22 mmol), solvent (1 mL), rt, 16 h.

^*b*^Yields of isolated **3a** after flash column chromatography.

^*c*^Ratio based on isolated yields.

^*d*^Determined by ^1^H NMR.

^*e*^The ee value was determined by HPLC on a chiral stationary phase.

^*f*^Addition of 4 Å MS. DIPEA = *N*,*N*-diisopropylethylamine, TMEDA = tetramethylethylenediamine, DABCO = 1,4-diazabicyclo[2.2.2]octane, DBU = 1,8-diazabicyclo-[5.4.0]undec-7-ene, Mes = 2,4,6-trimethylphenyl, TBDPS = *tert*-butyldiphenylsilyl, and TIPS = triisopropylsilyl.

Next we investigated the substrate scope of this protocol on a 0.5 mmol scale. As shown in Table 2, a wide range of 2-benzothiazolimines 1 with diverse electronic and steric properties were first explored. The use of 2-benzothiazolimines **1a** afforded the desired **3a** in 63% yield with >20 : 1 d.r. and 90% ee (Table 2, **3a**). Electron-donating substituents such as 4-Me and 4-OMe on the Ar group reduced the electrophilicity of the imine carbon, which led to lower yields and even the diasteroselectivity in **3c** (Table 2, **3b**,**c**). In the case of electron-withdrawing groups such as 4-Br, 4-Cl and 2-Cl, the reactions gave the desired cycloadducts **3d–f** in good yields and with good to excellent diastereo- and enantioselectivities. The introduction of a heterocyclic furyl group on the Ar position gave compatible results, affording the corresponding product **3g** in 69% yield, 11 : 1 d.r. and 93% ee under an elevated temperature. Several electron-donating and electron-withdrawing substituents such as R^1^ were also investigated, yielding the cycloadducts **3h–k** in good yields and excellent stereoselectivities. We then varied the α-chloroaldehyde moiety. An aliphatic linear α-chloroaldehyde reacted smoothly with a slight loss of yield and with a reasonable ee value (Table 2, **3l**). When a *para*-nitrophenyl group instead of Ph as R^2^ was used, a better result in terms of yield and ee was obtained (Table 2, **3m**).

**Table 2:** Substrate scope^*a*^

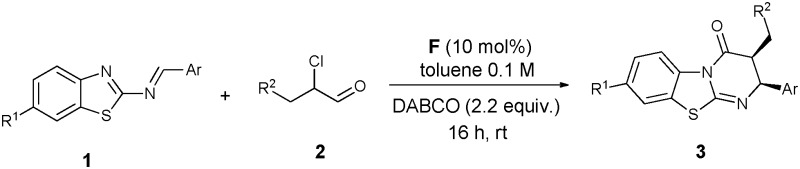						
**3**	R^1^	Ar	R^2^	Yield^*b*^ (%)	dr^*c*^	ee^*d*^ (%)
**a**	H	Ph	Ph	63	>20 : 1	90
**b** ^*e*^	H	4-MePh	Ph	49	>20 : 1	99
**c** ^*f*^	H	4-MeOPh	Ph	34	4 : 1	87
**d**	H	4-BrPh	Ph	56	11 : 1	91
**e**	H	4-ClPh	Ph	61	11 : 1	89
**f**	H	2-ClPh	Ph	60	>20 : 1	97
**g** ^*f*^	H	2-Furyl	Ph	69	11 : 1	93
**h** ^*e*^	Me	Ph	Ph	64	20 : 1	93
**i** ^*g*^	MeO	Ph	Ph	56	>20 : 1	92
**j**	Cl	Ph	Ph	78	10 : 1	91
**k**	F	Ph	Ph	71	17 : 1	89
**l**	H	Ph	*n*-Propyl	51	17 : 1	87
**m**	H	Ph	4-NO_2_C_6_H_4_	69	13 : 1	92

^*a*^Reaction conditions: **1** (0.5 mmol), **2** (1.0 mmol), **F** (0.05 mmol), DABCO (1.1 mmol), toluene (5 mL), 4 Å MS, rt, 16 h.

^*b*^Yield of isolated **3** after flash column chromatography.

^*c*^Determined by ^1^H NMR.

^*d*^The ee value was determined by HPLC on a chiral stationary phase.

^*e*^The reaction time is 24 h.

^*f*^Performed at 40 °C.

^*g*^The reaction time is 48 h.

The relative configuration of the major diastereomer **3a** was determined by NOE measurements (see ESI†), which is in accordance with the absolute configuration of compound **3e** determined by X-ray crystal structure analysis (Fig. 2).^10^

**Fig. 2:**
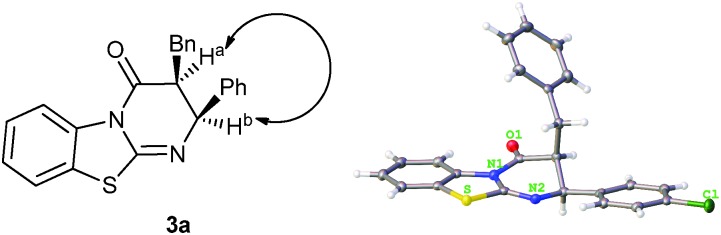
Determination of the relative configuration by NOE (**3a**) and of the absolute configuration by X-ray crystal structure analysis (**3e**).

We then tried to extend the substrate scope by employing a 2-indolyl group on the Ar position. In this case, after the Mannich reaction, there are three nucleophilic N-sites for the subsequent lactamization. Interestingly, only the *trans*-pyrrolo[1,2*-a*]indolone **3n** was obtained *via* cyclization of the indole N-anion with the acylazolium intermediate with acceptable yield (45%) and excellent stereoselectivity (93% ee, 11 : 1 d.r.) (Scheme 2).

**Scheme 2:**
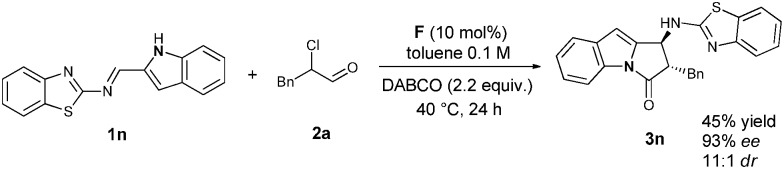
Asymmetric synthesis of **3n** *via* an NHC-catalyzed [2+3] annulation.

With the substrate scope and stereochemical outcome in hand, we propose a plausible catalytic cycle *via* a stepwise reaction sequence. As shown in Scheme 3, the nucleophilic addition of NHC to α-chloroaldehyde gives rise to the intermediate **I**, followed by base assisted HCl-elimination to provide the enolate species **II**. This azolium-enolate then reacts on its *Re*-face with 2-benzothiazolimine **1** *via* a Mannich reaction to afford the adduct **III** with *cis* selectivity. Finally, the benzothiazole N-anion then cyclizes with the acylazolium intermediate liberating the NHC catalyst and producing the desired benzothiazolopyrimidinone **3** in *cis* configuration.

**Scheme 3:**
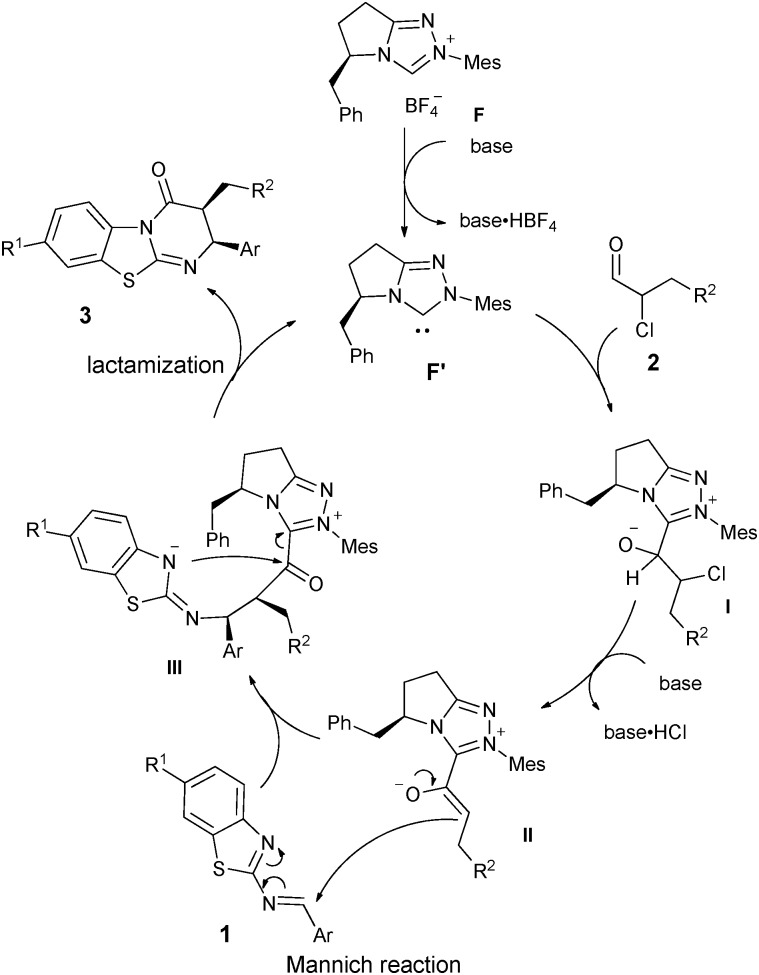
Proposed mechanism of the reaction.

In conclusion, we have developed an asymmetric NHC-organocatalyzed annulation of 2-benzothiazolimines with α-chloroaldehydes, producing the desired benzothiazolo-pyrimidinones in moderate to good yields with excellent regio- and stereoselectivities. Particularly noteworthy is the reaction of indolyl-bound 2-benzothiazolimine with 2-chloro-3-phenylpropanal. This version of the protocol leads to the formation of pyrrolo[1,2*-a*]indolone with high regioselectivity and excellent stereoselectivity. Further applications of this protocol in the scope and application are ongoing in our laboratory.

We are grateful for the financial support from the European Research Council (ERC Advanced Grant 320493 “DOMINOCAT”). Q. Ni and X. Song thank the China Scholarship Council for a fellowship.
