# Supplementary material for: Regio- and stereoselective synthesis of benzothiazolo-pyrimidinones via an NHC-catalyzed Mannich/lactamization domino reaction
Source: Chem Commun (Camb). 2014 Dec 5;51(7):1263–6. doi: 10.1039/c4cc08594a (PMC4612130; doi:10.1039/c4cc08594a)
Supplement: Supplementary file 1 [file CC-051-C4CC08594A-s001.pdf]

Electronic Supplementary Material (ESI) for ChemComm.
This journal is © The Royal Society of Chemistry 2014

# Content

1. General information ........................................................................................................2

2. General procedure............................................................................................................2

3. Characterization Data .......................................................................................................3

4. The NOESY spectrum of 3a ..............................................................................................9

5. References ........................................................................................................................9

6. Copies of NMR spectra and HPLC measurements of the products *cis*-3 (plus minor trans-isomer).........................................................................................................................10

## 1. General information

- All reactions were carried out under argon. THF and toluene were distilled over Solvona ®. Dichloromethane was distilled over calcium hydride. All other chemicals were used without further purification.
- Triazolium salts **A**-**G** were prepared according to known literature procedures.[1] Benzothiazolimines **1** were prepared from benzo[*d*]thiazol-2-amine.[2] α-Chloroaldehydes were synthesized form aldehydes.[3] Racemic samples were prepared by using triazolium salt **A** as catalyst, except **3l**, which was prepared by manully mixing **3l** and *ent*-**3l**.
- Chromatographic purification of the products was performed on Merck silica gel 60, particle size 0.040-0.063 mm (230-240 mesh, flash).
- Analytical TLC: SIL G-25 UV254 from MACHEREY&NAGEL. Visualization of the developed TLC plates was performed with ultraviolet irradiation (254 nm).
- Microanalyses were performed with a Vario EL element analyzer.
- Mass spectra and high resolution mass spectra were acquired on a Finnigan MAT 95 (EI/CI) or on a ThermoFisher Scientific LTQ Orbitrap XL (ESI).
- IR spectra were taken on a Perkin-Elmer FT-IR Spectrum 100 using a Diamant/KRS5 ATR. Evaluation was done using the supplementary software. The absorption bands are given in wave numbers ($cm^{-1}$).
- $^{1}H$- and $^{13}C$ NMR were recorded at ambient temperature on VNMRS 600 and Inova 400 instruments. The chemical shifts are reported in ppm downfield of tetramethylsilane (TMS) and referenced to residual solvent peaks resonance as internal standard. The order of citation in parentheses is a) multiplicity (s = singlet, d = doublet, dd = doublet of doublet, t = triplet, q = quartet, td = triplet of doublet, m = multiplet), b) coupling constants, c) number of protons, and d) assignment. Coupling constants (J) are reported in Hertz (Hz).
- Analytical HPLC was performed on a Hewlett-Packard 1100 Series instrument using chiral stationary phases (Daicel IC, Daicel IA, Daicel AD, Daicel OJ ).
- Optical rotation values were measured on a Perkin-Elmer 241 polarimeter.

## 2. General procedure

To a dried and argon-filled Schlenk flask was added (*E*)- *N*-arylidenebenzo[d]thiazol-2-amine (**1**) (0.5 mmol, 1.0 equiv.), α-chloroaldehyde **2** (1.0 mmol, 2.0 equiv.), triazolium salt **F** (0.05 mmol, 10 mol%) and DABCO (1.1 mmol, 2.2 equiv.) in toluene (5 ml). The mixture was stirred at room temperature and monitored by TLC until completion of the reaction. The solution was directly purified by flash chromatography on silica gel (pentane/$Et_2O$ 10:1) to afford the products **3a-n**.

## 3. Characterization Data

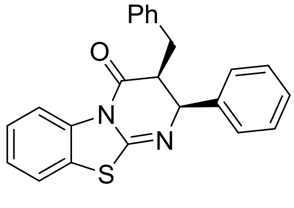

The compound **3a** was prepared according to the general procedure. The product was obtained as a colorless solid (116 mg, 63% yield). The ee (90%) was measured by HPLC using a chiral stationary phase [Daicel IC, *n*-heptane:*i*-PrOH = 9:1, 0.5 mL/min), $t_R$ = 8.85 min (major), 10.43 min (minor)], $[\alpha]_D^{22}$ = +163.8 (c = 1.0, $CHCl_3$). Melting point: 154-155 °C.

$^1$H NMR (600 MHz, Chloroform-*d*) δ 8.26 (dd, *J* = 8.1, 1.2 Hz, 1H), 7.39 (d, *J* = 4.3 Hz, 4H), 7.36 - 7.31 (m, 2H), 7.29 - 7.26 (m, 1H), 7.24 - 7.15 (m, 4H), 7.02 - 6.88 (m, 2H), 5.03 (d, *J* = 5.8 Hz, 1H), 3.31 (ddd, *J* = 7.9, 6.6, 5.8 Hz, 1H), 3.00 (dd, *J* = 14.5, 7.9 Hz, 1H), 2.56 (dd, *J* = 14.5, 6.6 Hz, 1H);

$^{13}$C NMR (151 MHz, Chloroform-*d*) δ 169.62, 155.83, 138.05, 137.69, 135.62, 128.82 (4C), 128.39 (2C), 127.90, 127.36 (2C), 126.55, 126.50, 125.62, 123.16, 121.75, 116.76, 62.47, 47.38, 31.04;

MS (EI, 70 eV) m/z (%): 370 [$M^+$] (95), 279 (33), 239 (28), 238 (58), 237 (100);

IR (ATR): 3034, 2928, 1709, 1642, 1453, 1301, 1176, 1014, 913, 865, 745, 697 $cm^{-1}$;

Anal. calcd. for $C_{23}H_{18}N_2OS$ (370) C, 74.57; H, 4.90; N, 7.56 found: C, 74.42; H, 4.91; N, 7.44.

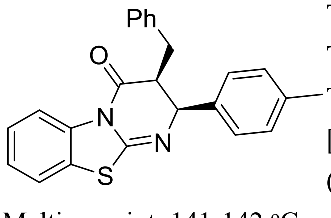

The compound **3b** was prepared according to the general procedure. The product was obtained as a colorless solid (94 mg, 49% yield). The ee (99%) was measured by HPLC using a chiral stationary phase [Daicel IA, *n*-heptane:EtOH = 7:3, 0.7 mL/min), $t_R$ = 11.14 min (minor), 11.91 min (major)], $[\alpha]_D^{22}$ = +241.7 (c = 1.0, $CHCl_3$). Melting point: 141-142 °C.

$^1$H NMR (600 MHz, Chloroform-*d*) δ 8.26 (dd, *J* = 8.2, 1.2 Hz, 1H), 7.33 (dd, *J* = 7.7, 1.4 Hz, 1H), 7.25 (ddd, *J* = 6.8, 4.5, 2.1 Hz, 3H), 7.23 - 7.15 (m, 6H), 6.98 (dd, *J* = 6.9, 1.8 Hz, 2H), 4.97 (d, *J* = 5.9 Hz, 1H), 3.30 (td, *J* = 7.3, 6.2 Hz, 1H), 3.02 (dd, *J* = 14.4, 7.6 Hz, 1H), 2.56 (dd, *J* = 14.5, 6.8 Hz, 1H), 2.36 (s, 3H);

$^{13}$C NMR (151 MHz, Chloroform-*d*) δ 169.71, 155.62, 138.20, 137.61, 135.64, 134.51, 129.49 (2C), 128.83 (2C), 128.38 (2C), 127.25 (2C), 126.51, 126.46, 125.57, 123.19, 121.73, 116.72, 62.29, 47.33, 31.02, 21.13;

MS (EI, 70 eV) m/z (%):384 [$M^+$] (19), 293 (24), 252 (45), 251 (100), 131 (22), 91 (78);

IR (ATR): 3025, 2930, 1713, 1641, 1506, 1456, 1301, 1174, 1018, 923, 857, 803, 741, 697 $cm^{-1}$;

HRMS (ESI): calcd for $C_{24}H_{20}N_2OS$ $[M+H]^+$: 385.1369; found: 385.1371.

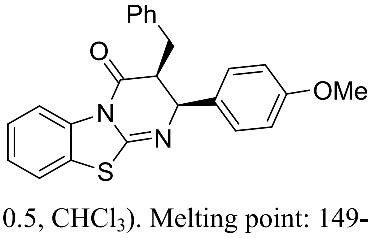

The compound **3c** was prepared according to the general procedure. The product was obtained as a yellow solid (68 mg, 34% yield). The ee (87%) was measured by HPLC using a chiral stationary phase [Daicel IA, *n*-heptane:EtOH = 7:3, 0.7 mL/min), $t_R$ = 14.96 min (minor), 17.15 min (major)], $[\alpha]_D^{22}$ = +167.4 (c = 0.5, $CHCl_3$). Melting point: 149-150 °C.

$^1$H NMR (600 MHz, Chloroform-*d*) δ 8.26 (dt, *J* = 8.2, 1.2 Hz, 1H), 7.29 - 7.24 (m, 4H), 7.21 (tt, *J* = 9.9, 2.7 Hz, 4H), 7.03 - 6.96 (m, 2H), 6.95 - 6.87 (m, 2H), 4.95 (d, *J* = 5.9 Hz, 1H), 3.82 (s, 3H), 3.30 (q, *J* = 6.9 Hz, 1H), 3.05 (dd, *J* = 14.5, 7.6 Hz, 1H), 2.55 (dd, *J* = 14.5, 6.9 Hz, 1H);

$^{13}C$ NMR (151 MHz, Chloroform-*d*) δ 169.72, 159.25, 155.57, 138.21, 135.63, 131.35, 128.82 (2C), 128.53(2C), 128.41 (2C), 126.52, 126.48, 125.58, 123.17, 121.74, 116.71, 114.19 (2C), 61.97, 55.28, 47.35, 31.01;
MS (EI, 70 eV) m/z (%):400 [$M^+$] (22), 308 (35), 268 (88), 267 (96), 135 (100), 91 (43);
IR (ATR): 3064, 3024, 2928, 2842, 1715, 1638, 1509, 1455, 1293, 1246, 1173, 1028, 911, 826, 743, 697 $cm^{-1}$;
HRMS (ESI): calcd for $C_{24}H_{20}N_2O_2S$ $[M+Na]^+$: 423.1138; found: 423.1131.

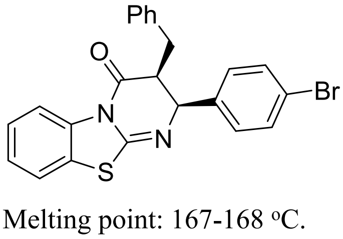

The compound **3d** was prepared according to the general procedure. The product was obtained as a colorless solid (125 mg, 56% yield). The ee (91%) was measured by HPLC using a chiral stationary phase [Daicel IA, *n*-heptane:EtOH = 7:3, 0.7 mL/min), $t_R$ = 12.98 min (minor), 15.30 min (major)], $[\alpha]_D^{22}$ = +164.5 (c = 1.0, $CHCl_3$). Melting point: 167-168 °C.
$^1H$ NMR (400 MHz, Chloroform-*d*) δ 8.30 - 8.10 (m, 1H), 7.59 - 7.07 (m, 10H), 6.92 (dd, *J* = 7.4, 2.0 Hz, 2H), 4.98 (d, *J* = 5.6 Hz, 1H), 3.25 (ddd, *J* = 8.0, 6.5, 5.6 Hz, 1H), 2.93 (dd, *J* = 14.4, 8.0 Hz, 1H), 2.52 (dd, *J* = 14.4, 6.6 Hz, 1H);
$^{13}C$ NMR (101 MHz, Chloroform-*d*) δ 169.32, 156.21, 137.62, 136.92, 135.51, 131.88 (2C), 129.04 (2C), 128.76 (2C), 128.43 (2C), 126.65, 126.57, 125.74, 123.04, 121.78, 121.75, 116.80, 61.75 , 47.24, 31.03;
MS (EI, 70 eV) m/z (%):450 [M+2] (50), 448 [$M^+$] (44), 359 (35), 357 (34), 318 (76), 317 (100), 316 (67), 315 (78), 135 (24), 91 (19);
IR (ATR): 3027, 2926, 1714, 1638, 1582, 1485, 1458, 1299, 1180, 1004, 914, 858, 801, 746, 694 $cm^{-1}$;
HRMS (ESI): calcd for $C_{23}H_{17}BrN_2OS$ $[M+Na]^+$: 471.0137; found: 471.0138.

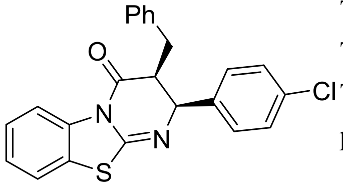

The compound **3e** was prepared according to the general procedure. The product was obtained as a colorless solid (123 mg, 61% yield). The ee (89%) was measured by HPLC using a chiral stationary phase [Daicel AD, *n*-heptane:EtOH = 9:1, 1.0 mL/min), $t_R$ = 12.07min (minor), 14.44 min (major)], $[\alpha]_D^{22}$ = +166.0 (c = 1.0, $CHCl_3$). Melting point: 162-163 °C.
$^1H$ NMR (600 MHz, Chloroform-*d*) δ 8.24 (dd, *J* = 8.1, 1.2 Hz, 1H), 7.49 - 7.12 (m, 10H), 7.04 - 6.83 (m, 2H), 5.01 (d, *J* = 5.6 Hz, 1H), 3.35 - 3.20 (m, 1H), 2.95 (dd, *J* = 14.4, 8.0 Hz, 1H), 2.53 (dd, *J* = 14.5, 6.6 Hz, 1H);
$^{13}C$ NMR (151 MHz, Chloroform-*d*) δ 169.35, 156.24, 137.66, 136.38, 135.53, 133.65, 128.95 (2C), 128.77 (2C), 128.72 (2C), 128.44 (2C), 126.66, 126.59, 123.06, 125.75, 121.79, 116.81, 61.71, 47.30, 31.03;
MS (EI, 70 eV) m/z (%): 406 [M+2] (33), 404 [$M^+$] (83), 313 (35), 274 (30), 272 (89), 270 (100), 135 (33), 91 (90);
IR (ATR): 3062, 2933, 1714, 1638, 1463, 1301, 1177, 1093, 1009, 923, 852, 808, 740, 696 $cm^{-1}$;
Anal. calcd. for $C_{23}H_{17}ClN_2OS$ (404) C, 68.22; H, 4.23; N, 6.92 found: C, 68.42; H, 4.15; N, 6.56.

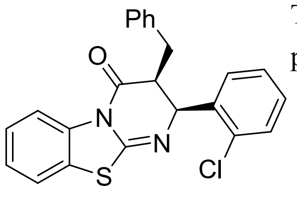

The compound **3f** was prepared according to the general procedure. The product was obtained as a colorless solid (121 mg, 60% yield). The ee

(97%) was measured by HPLC using a chiral stationary phase [Daicel OJ, *n*-heptane:EtOH = 7:3, 0.7 mL/min), $t_R$ = 13.20 min (major), 17.14 min (minor)], $[\alpha]_D^{22}$ = +253.8 (c = 1.0, $CHCl_3$). Melting point: 170-171 °C.

$^1$H NMR (400 MHz, Chloroform-*d*) δ 7.88 - 7.66 (m, 2H), 7.42 - 7.35 (m, 2H), 7.33 - 7.24 (m, 4H), 7.23 - 7.16 (m, 3H), 7.07 - 6.93 (m, 2H), 5.93 (d, *J* = 6.1 Hz, 1H), 4.18 (ddd, *J* = 9.0, 6.8, 6.1 Hz, 1H), 2.75 (dd, *J* = 14.6, 9.0 Hz, 1H), 2.56 (dd, *J* = 14.6, 6.8 Hz, 1H);

$^{13}$C NMR (101 MHz, Chloroform-*d*) δ 166.70, 149.30, 137.18, 133.28, 132.24, 131.89, 129.79, 129.51, 128.69 (2C), 128.38 (2C), 127.75, 126.97, 126.58, 126.32, 124.32, 122.68, 122.00, 121.36, 58.69, 56.38, 31.66;

MS (EI, 70 eV) m/z (%):406 [M+2] (7), 404 [M$^+$] (19), 237 (100), 91 (22);

IR (ATR): 3061, 2934, 1765, 1597, 1520, 1441, 1355, 1274, 1033, 899, 749, 668 cm$^{-1}$;

HRMS (ESI): calcd for $C_{23}H_{17}ClN_2OS$ [M+Na]$^+$: 427.0642; found: 427.0637.

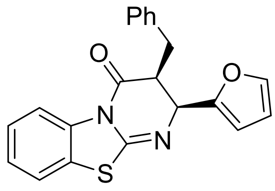


The compound **3g** was prepared according to the general procedure. The product was obtained as a colorless solid (124 mg, 69% yield). The ee (93%) was measured by HPLC using a chiral stationary phase [Daicel AD, *n*-heptane:EtOH = 7:3, 0.7 mL/min), $t_R$ = 10.76 min (minor), 13.45 min (major)], $[\alpha]_D^{22}$ = +336.9 (c = 1.0, $CHCl_3$). Melting point: 52-53 °C.

$^1$H NMR (400 MHz, Chloroform-*d*) δ 8.41 - 8.27 (m, 1H), 7.34 (s, 1H), 7.32 - 7.26 (m, 3H), 7.25 - 7.17 (m, 3H), 7.12 (d, *J* = 7.0 Hz, 2H), 6.37 - 6.24 (m, 2H), 4.82 (d, *J* = 6.5 Hz, 1H), 3.41 (dd, *J* = 14.3, 5.1 Hz, 1H), 3.32 (ddd, *J* = 9.1, 6.5, 5.1 Hz, 1H), 2.42 (dd, *J* = 14.3, 9.0 Hz, 1H);

$^{13}$C NMR (101 MHz, Chloroform-*d*) δ 169.06, 157.52, 150.43, 142.81, 138.30, 135.79, 128.85 (2C), 128.54 (2C), 126.63, 126.50, 125.47, 123.01, 121.67, 116.72, 110.22, 109.12, 55.95, 45.24, 30.99;

MS (EI, 70 eV) m/z (%):362 [M+2] (25), 361 [M+1] (72), 360 [M$^+$] (100), 269 (50), 228 (71), 200 (37), 187 (30), 91 (26);

IR (ATR): 3028, 2927, 1719, 1633, 1462, 1301, 1172, 817, 740, 703 cm$^{-1}$;

HRMS (ESI): calcd for $C_{21}H_{17}N_2O_2S$ [M+H]$^+$: 361.1005; found: 361.1001.

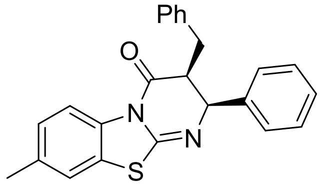


The compound **3h** was prepared according to the general procedure. The product was obtained as a colorless solid (123 mg, 64% yield). The ee (93%) was measured by HPLC using a chiral stationary phase [Daicel IC, *n*-heptane:*i*-PrOH = 9:1, 0.5 mL/min), $t_R$ = 10.54 min (major), 12.31 min (minor)], $[\alpha]_D^{22}$ = +150.9 (c = 1.0, $CHCl_3$). Melting point: 153-154 °C.

$^1$H NMR (400 MHz, Chloroform-*d*) δ 8.11 (d, *J* = 8.4 Hz, 1H), 7.41 – 7.27 (m, 5H), 7.22 – 7.10 (m, 4H), 7.09 – 7.00 (m, 1H), 7.00 – 6.87 (m, 2H), 5.00 (d, *J* = 5.8 Hz, 1H), 3.28 (dt, *J* = 7.9, 6.3 Hz, 1H), 2.97 (dd, *J* = 14.4, 7.9 Hz, 1H), 2.54 (dd, *J* = 14.4, 6.6 Hz, 1H), 2.35 (s, 3H);

$^{13}$C NMR (101 MHz, Chloroform-*d*) δ 169.44, 156.08, 138.10, 137.77, 135.65, 133.36, 128.81 (2C), 128.78 (2C), 128.36 (2C), 127.84, 127.35 (2C), 127.12, 126.51, 123.02, 122.09, 116.46, 62.47, 47.32, 31.03, 21.12;

MS (EI, 70 eV) m/z (%):385 [M+1] (37), 384 [M$^+$] (87), 293 (41), 253 (30), 252 (84), 251 (100), 91 (25);

IR (ATR): 3029, 2923, 1718, 1633, 1476, 1297, 1170, 910, 873, 819, 743, 699 cm$^{-1}$;

HRMS (ESI): calcd for $C_{24}H_{20}N_2OS$ [M+H]$^+$: 385.1369; found: 385.1372.

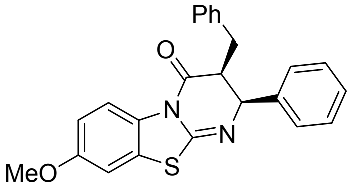


The compound **3i** was prepared according to the general procedure. The product was obtained as a colorless solid (111 mg, 56% yield). The ee (92%) was measured by HPLC using a chiral stationary phase [Daicel AD, *n*-heptane:EtOH = 7:3, 0.7 mL/min), $t_R$ = 13.39 min (minor), 16.40 min (major)], $[\alpha]_D^{22}$ = +131.0 (c = 1.0, $CHCl_3$).

Melting point: 144-145 °C.

$^1$H NMR (600 MHz, Chloroform-*d*) δ 8.16 (d, *J* = 9.0 Hz, 1H), 7.41 - 7.28 (m, 5H), 7.22 - 7.14 (m, 3H), 6.97 - 6.93 (m, 2H), 6.89 (d, *J* = 2.6 Hz, 1H), 6.78 (dd, *J* = 9.0, 2.6 Hz, 1H), 5.02 (d, *J* = 5.8 Hz, 1H), 3.82 (d, *J* = 0.8 Hz, 3H), 3.28 (ddd, *J* = 7.9, 6.6, 5.7 Hz, 1H), 2.97 (dd, *J* = 14.5, 7.9 Hz, 1H), 2.55 (dd, *J* = 14.4, 6.6 Hz, 1H);

$^{13}$C NMR (151 MHz, Chloroform-*d*) δ 169.25, 157.47, 156.04, 138.10, 137.80, 128.83 (2C), 128.79 (2C), 128.36 (2C), 127.85, 127.36 (2C), 126.52, 124.44, 117.54, 111.70, 107.57, 104.56, 62.55, 55.71, 47.23, 31.03;

MS (EI, 70 eV) m/z (%): 401 [M+1] (32), 400 [M$^+$] (93), 268 (100), 267 (89), 253 (22), 91 (18);

IR (ATR): 3061, 3027, 2964, 2841, 1713, 1641, 1595, 1479, 1328, 1255, 1175, 1027, 916, 881, 827, 741, 696 cm$^{-1}$;

Anal. calcd. for $C_{24}H_{20}N_2O_2S$ (400) C, 71.98; H, 5.03; N, 6.99 found: C, 71.64; H, 5.17; N, 6.59.

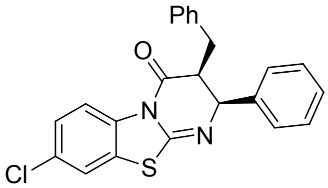


The compound **3j** was prepared according to the general procedure. The product was obtained as a colorless solid (157 mg, 78% yield). The ee (91%) was measured by HPLC using a chiral stationary phase [Daicel IC, *n*-heptane:EtOH = 7:3, 0.5 mL/min), $t_R$ = 18.59 min (minor), 20.67 min (major)], $[\alpha]_D^{22}$ = +109.1 (c = 1.0, $CHCl_3$).

Melting point: 63-64 °C.

$^1$H NMR (400 MHz, Chloroform-*d*) δ 8.16 (d, *J* = 8.8 Hz, 1H), 7.42 - 7.12 (m, 10H), 7.01 - 6.87 (m, 2H), 5.02 (d, *J* = 5.8 Hz, 1H), 3.29 (ddd, *J* = 7.9, 6.5, 5.7 Hz, 1H), 2.95 (dd, *J* = 14.4, 7.9 Hz, 1H), 2.55 (dd, *J* = 14.4, 6.6 Hz, 1H);

$^{13}$C NMR (101 MHz, Chloroform-*d*) δ 169.44, 155.12, 137.82, 137.47, 134.14, 131.00, 128.86 (2C), 128.80 (2C), 128.41 (2C), 127.98, 127.28 (2C), 126.63, 126.59, 124.95, 121.64, 117.46, 62.53, 47.29, 31.03;

MS (EI, 70 eV) m/z (%): 406 [M+2] (10), 404 [M$^+$] (27), 274 (32), 273 (57), 272 (81), 271 (100), 91 (90);

IR (ATR): 3063, 3028, 2927, 1720, 1640, 1577, 1459, 1291, 1176, 1144, 1086, 908, 865, 816, 736, 697 cm$^{-1}$;

HRMS (ESI): calcd for $C_{23}H_{17}ClN_2OS$ $[M+Na]^+$: 427.0642; found: 427.0640.

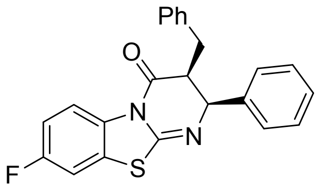


The compound **3k** was prepared according to the general procedure. The product was obtained as a colorless solid (138 mg, 71% yield). The ee (89%) was measured by HPLC using a chiral stationary phase [Daicel IA, *n*-heptane:EtOH = 7:3, 0.5 mL/min), $t_R$ = 16.22 min (minor), 17.76 min (major)], $[\alpha]_D^{22}$ = +126.4 (c = 1.0, $CHCl_3$).

Melting point: 55-56 °C.

$^1$H NMR (400 MHz, Chloroform-*d*) δ 8.21 (dd, *J* = 9.1, 4.8 Hz, 1H), 7.40 - 7.31 (m, 5H), 7.22 - 7.13

(m, 3H), 7.06 (dd, *J* = 7.7, 2.6 Hz, 1H), 6.94 (dd, *J* = 7.2, 1.6 Hz, 3H), 5.02 (d, *J* = 5.8 Hz, 1H), 3.29 (ddd, *J* = 7.8, 6.6, 5.8 Hz, 1H), 2.95 (dd, *J* = 14.4, 7.8 Hz, 1H), 2.55 (dd, *J* = 14.3, 6.6 Hz, 1H);

$^{13}$C NMR (101 MHz, Chloroform-*d*) δ 169.40, 160.08 (d, *J* = 246.4 Hz), 155.46, 137.71 (d, *J* = 35.8 Hz), 131.88, 131.40, 128.85 (2C), 128.81 (2C), 128.40 (2C), 127.95, 127.31 (2C), 126.60, 124.90 (d, *J* = 9.9 Hz), 117.74 (d, *J* = 8.3 Hz), 113.24 (d, *J* = 22.9 Hz), 109.31 (d, *J* = 27.3 Hz), 62.55, 47.22, 31.03;

MS (EI, 70 eV) m/z (%):388 [M$^+$] (66), 257 (26), 256 (86), 255 (100);

IR (ATR): 3062, 3030, 2928, 2856, 1719, 1640, 1592, 1471, 1221, 1164, 899, 852, 749, 698 cm$^{-1}$;

HRMS (ESI): calcd for $C_{23}H_{17}FN_2OS$ [M+Na]$^+$: 411.0938; found: 427.0934.

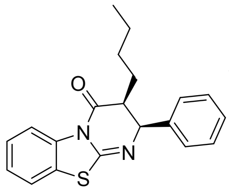

The compound **3l** was prepared according to the general procedure. The product was obtained as a yellow oil (86 mg, 51% yield). The ee (87%) was measured by HPLC using a chiral stationary phase [Daicel IC, *n*-heptane:*i*-PrOH = 9:1, 0.7 mL/min), $t_R$ = 4.90 min (minor), 5.41 min (major)], $[\alpha]_D^{22}$ = +47.9 (c = 1.0, $CHCl_3$).

$^1$H NMR (400 MHz, Chloroform-*d*) δ 8.32 (dd, *J* = 8.1, 1.3 Hz, 1H), 7.42 - 7.31 (m, 5H), 7.31 - 7.18 (m, 3H), 5.05 (d, *J* = 5.2 Hz, 1H), 2.93 - 2.78 (m, 1H), 1.57 - 1.46 (m, 1H), 1.30 - 1.16 (m, 4H), 0.92 - 0.84 (m, 1H), 0.75 (t, *J* = 7.1 Hz, 3H);

$^{13}$C NMR (101 MHz, Chloroform-*d*) δ 170.66, 155.64, 138.30, 135.78, 128.57 (2C), 127.51, 127.12 (2C), 126.45, 125.58, 123.32, 121.76, 116.90, 62.57, 45.77, 29.28, 24.15, 22.35, 13.71;

MS (EI, 70 eV) m/z (%):336 [M$^+$] (24), 334 (43), 292 (29), 291 (100), 254 (25), 237 (37), 105 (63), 77 (30);

IR (ATR): 3891, 3775, 3383, 3118, 2624, 2441, 2287, 2184, 2099, 1949, 1587, 1464, 1269, 1178, 1108, 933, 802, 720;

HRMS (ESI): calcd for $C_{20}H_{20}N_2OS$ [M+H]$^+$: 337.1369; found: 337.1369.

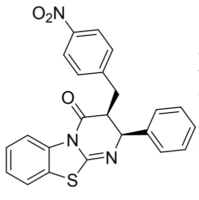

The compound **3m** was prepared according to the general procedure. The product was obtained as a colorless solid (143 mg, 69% yield). The ee (92%) was measured by HPLC using a chiral stationary phase [Daicel IC, *n*-heptane:EtOH = 7:3, 0.7 mL/min), $t_R$ = 27.73 min (major), 39.53 min (minor)], $[\alpha]_D^{22}$ = +110.4.1 (c = 0.5, $CHCl_3$). Melting point: 186-187 $^o$C.

$^1$H NMR (400 MHz, Chloroform-*d*) δ 8.21 (dd, *J* = 7.8, 1.6 Hz, 1H), 8.02 (d, *J* = 8.4 Hz, 2H), 7.42 – 7.30 (m, 6H), 7.25 (ddd, *J* = 9.0, 7.5, 1.6 Hz, 2H), 7.10 (d, *J* = 8.2 Hz, 2H), 5.05 (d, *J* = 5.7 Hz, 1H), 3.30 (dt, *J* = 8.4, 5.8 Hz, 1H), 2.96 (dd, *J* = 14.4, 8.3 Hz, 1H), 2.67 (dd, *J* = 14.4, 5.9 Hz, 1H);

$^{13}$C NMR (101 MHz, Chloroform-*d*) δ 168.95, 155.88, 146.71, 146.09, 137.39, 135.34, 129.74 (2C), 128.97 (2C), 128.13, 127.19 (2C), 126.65, 125.95, 123.57 (2C), 123.09, 121.88, 116.82, 62.44, 47.18, 31.27;

MS (EI, 70 eV) m/z (%):415 [M$^+$] (36), 238 (49), 237 (100);

IR (ATR): 3064, 2926, 2849, 1707, 1650, 1448, 1338, 1276, 1176, 1105, 854, 748, 702 cm$^{-1}$;

HRMS (ESI): calcd for $C_{23}H_{17}N_3O_3S$ [M+H]$^+$: 416.1063; found: 416.1063.

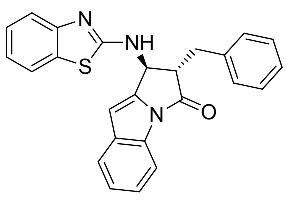

The compound **3n** was prepared according to the general procedure. The product was obtained as a colorless solid (91 mg, 45% yield). The ee (93%) was measured by HPLC using a chiral stationary phase [Daicel IA, *n*-heptane:EtOH = 7:3, 0.7 mL/min), $t_R$ = 10.10 min (minor), 12.19 min

(major)], $[\alpha]_D^{22}$ = -76.2 (c = 0.5, $CHCl_3$). Melting point: 78-79 $^oC$.

$^1H$ NMR (400 MHz, Chloroform-*d*) δ 8.07 (dq, *J* = 8.0, 0.9 Hz, 1H), 7.57 (ddd, *J* = 8.4, 7.2, 1.1 Hz, 2H), 7.47 (dt, *J* = 7.6, 1.0 Hz, 1H), 7.39 - 7.26 (m, 5H), 7.24 - 7.11 (m, 4H), 6.45 (d, *J* = 1.0 Hz, 1H), 5.51 (s, 1H), 5.43 (dd, *J* = 4.2, 1.4 Hz, 1H), 3.51 - 3.44 (m, 2H), 3.37 (dd, *J* = 15.4, 8.2 Hz, 1H);

$^{13}C$ NMR (101 MHz, Chloroform-*d*) δ 170.56, 164.85, 151.99, 141.23, 137.06, 134.60, 130.96, 130.37, 129.49 (2C), 128.70 (2C), 126.98, 126.08, 124.44, 124.39, 122.38, 121.32, 120.87, 119.66, 114.09, 102.52, 56.74, 52.09, 35.20;

MS (ESI) m/z (%):410.1314 [M+H], 432.1132 [M+Na];

IR (ATR): 2932, 2564, 2176, 2026, 1736, 1616, 1537, 1447, 1365, 1215, 1133, 1018, 935, 747, 699;

HRMS (ESI): calcd for $C_{25}H_{19}N_3OS$ $[M+Na]^+$: 432.1141; found: 432.1130.

## 4. The NOESY spectrum of 3a

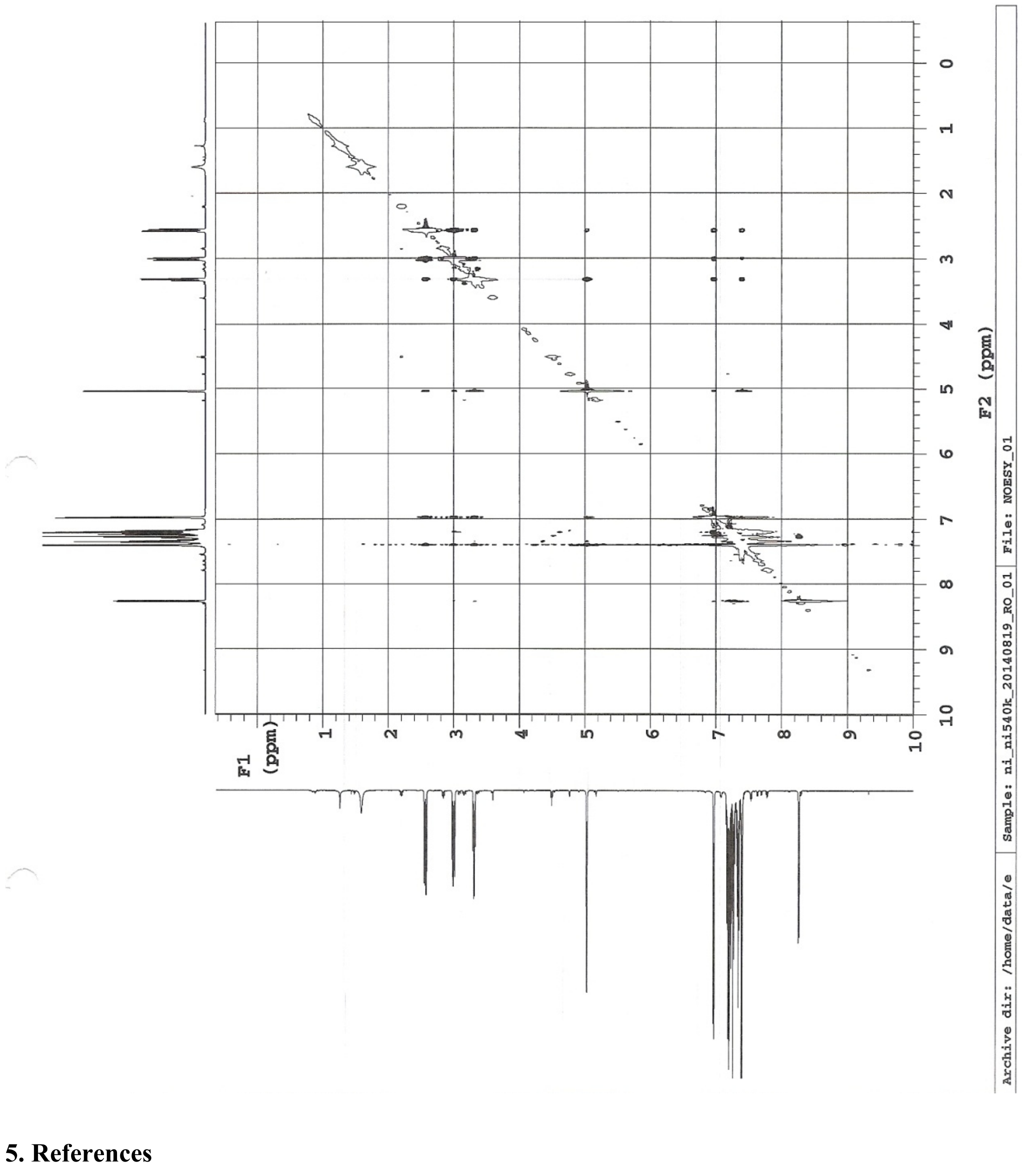


## 5. References

[1] a) N. E. Wurz, C. G. Daniliuc and F. Glorius, *Chem. Eur. J.*, 2012, **18**, 16297; b) H. U. Vora, S. P. Lathrop, N. T. Reynolds, M. S. Kerr, J. V. R. de Alaniz and T. Rovis, *Org. Synth.*, 2010, **87**, 350; c) J. R. Struble and J. W. Bode, *Org. Synth.*, 2010, **87**, 362; d) K. B. Ling and A. D. Smith, *Chem. Commun.*, 2011, **47**, 373.

[2] a) T.-S. Jin, M.-J. Yu, L.-B. Liu, Y. Zhao and T.-S. Li, *Synth. Commun.*, 2006, **36**, 2339.

[3] a) T. Borg, J. Danielsson and P. Somfai, *Chem. Commun.*, 2010, **46**, 1281; b) N. Halland, A. Braunton, S. Bachmann, M. Marigo and K. A. Jorgensen, *J. Am. Chem. Soc.*, 2004, **126**, 4790.

**6. Copies of NMR spectra and HPLC measurements of the products *cis*-3 (plus minor trans-isomer)**

$^{1}$H NMR of **3a**

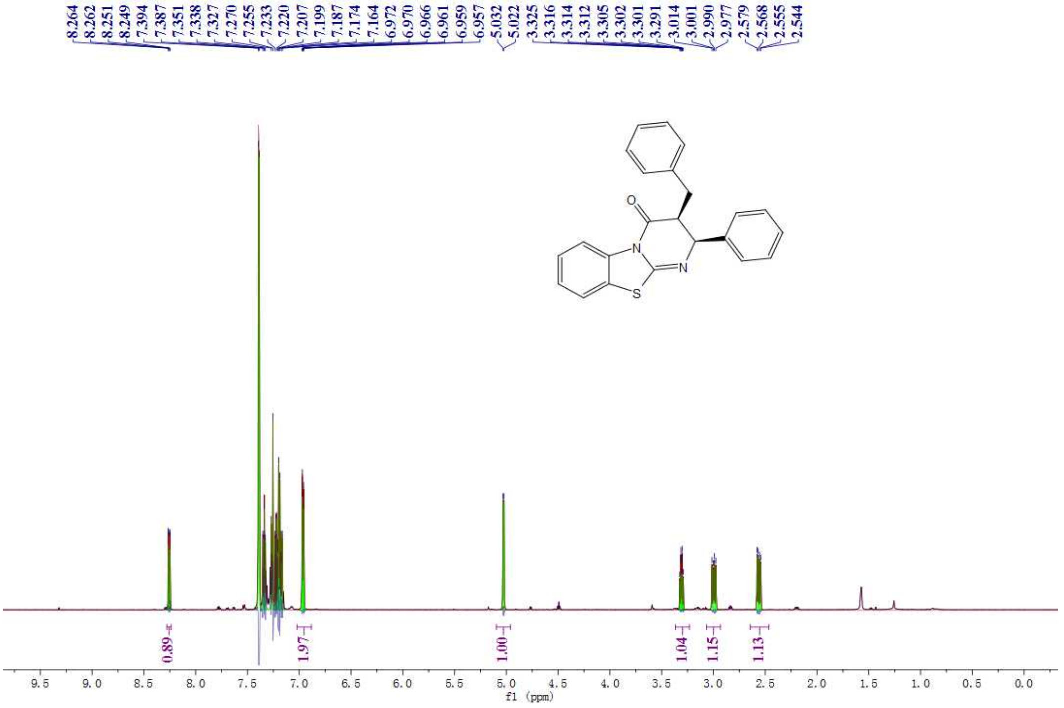


$^{13}$C NMR of **3a**

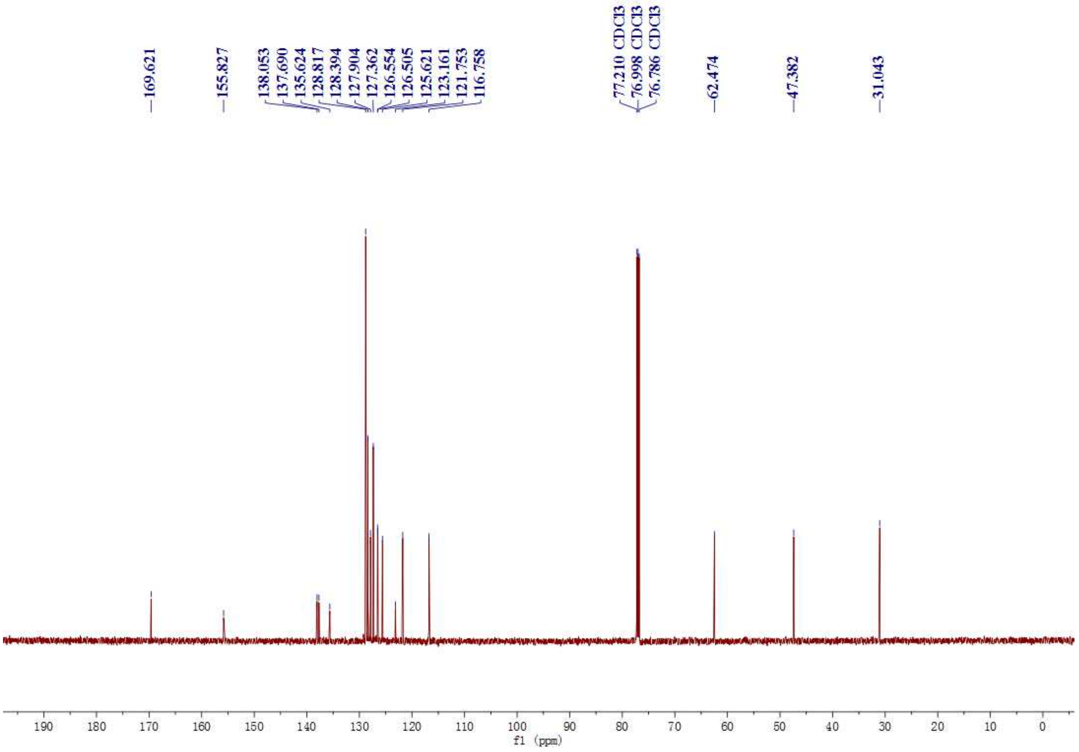

HPLC analysis: rac-**3a**

| | |
|---|---|
| Sample name: | Ni 61a rac |
| Data file: | C:\SNOOPY\NI\NI 61A RAC 1IC.D |
| Description: | Laufmittel: n-Heptan/IP 9:1 Die Probe ist DCM/LM gelöst. |
| Injection date: | 5/26/2014 8:55:07 AM |
| Acq. Analysis method: | CHIRALPAKIC1-6LNP.M |
| Column: | Chiralpak IC, (150 x 4,6) mm, 5μ, SN: IC00CD-QF015 |

Pressure at start: 21 bar Start flow: 0.500 ml/min Column oven: 29.99 °C

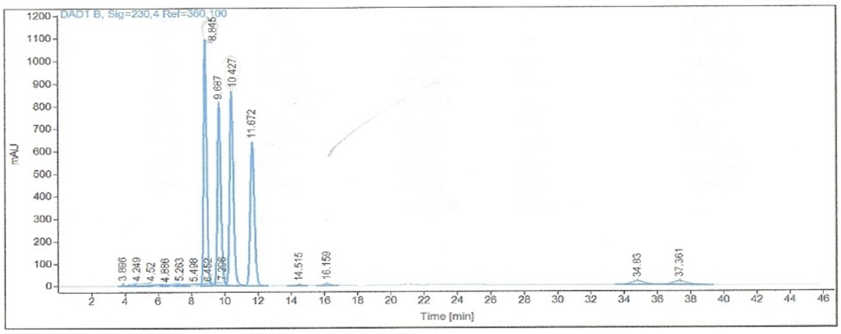


| RT [min] | Type | Area% | Area | Height | Width [min] |
|---|---|---|---|---|---|
| 3.90 | BV | 0.13 | 71.55 | 7.81 | 0.15 |
| 4.25 | VB | 0.10 | 53.30 | 7.22 | 0.11 |
| 4.52 | BV | 0.09 | 48.72 | 6.34 | 0.11 |
| 4.89 | VB | 0.04 | 18.76 | 1.54 | 0.16 |
| 5.26 | BV | 0.12 | 62.86 | 4.36 | 0.19 |
| 5.50 | VB | 0.04 | 18.99 | 2.52 | 0.11 |
| 6.45 | BB | 0.08 | 39.98 | 2.09 | 0.29 |
| 7.30 | BB | 0.26 | 137.81 | 6.73 | 0.33 |
| 8.85 | VB | 26.31 | 14009.02 | 1094.42 | 0.20 |
| 9.69 | BV | 21.86 | 11638.69 | 810.51 | 0.22 |
| 10.43 | VV | 25.64 | 13649.05 | 863.14 | 0.24 |
| 11.67 | VB | 20.99 | 11172.31 | 637.20 | 0.27 |
| 14.51 | BB | 0.19 | 101.32 | 3.64 | 0.42 |
| 16.16 | BB | 0.46 | 243.07 | 9.44 | 0.40 |
| 34.83 | BB | 1.86 | 987.69 | 17.28 | 0.88 |
| 37.36 | BB | 1.85 | 984.42 | 16.29 | 0.95 |
| | Sum | 100.00 | 53237.56 | | |

Enantioenriched **3a**

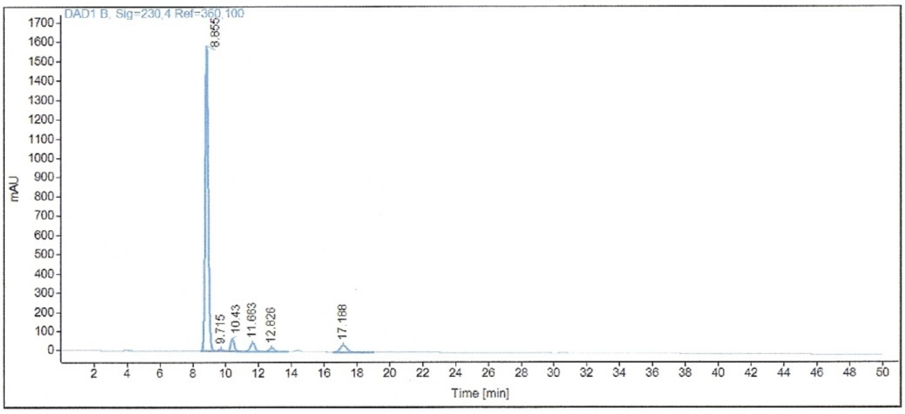


Name Ni 61 ac

| RT [min] | Type | Area% | Area | Height | Width [min] |
|---|---|---|---|---|---|
| 8.86 | BV | 85.42 | 20360.34 | 1585.23 | 0.20 |
| 9.71 | VB | 0.79 | 189.42 | 12.08 | 0.24 |
| 10.43 | BB | 4.33 | 1031.69 | 65.60 | 0.24 |
| 11.66 | BB | 3.63 | 865.41 | 45.11 | 0.29 |
| 12.83 | BB | 1.71 | 407.85 | 20.15 | 0.32 |
| 17.19 | BB | 4.11 | 980.72 | 34.84 | 0.44 |
| | Sum | 100.00 | 23835.43 | | |

$^1$H NMR of **3b**

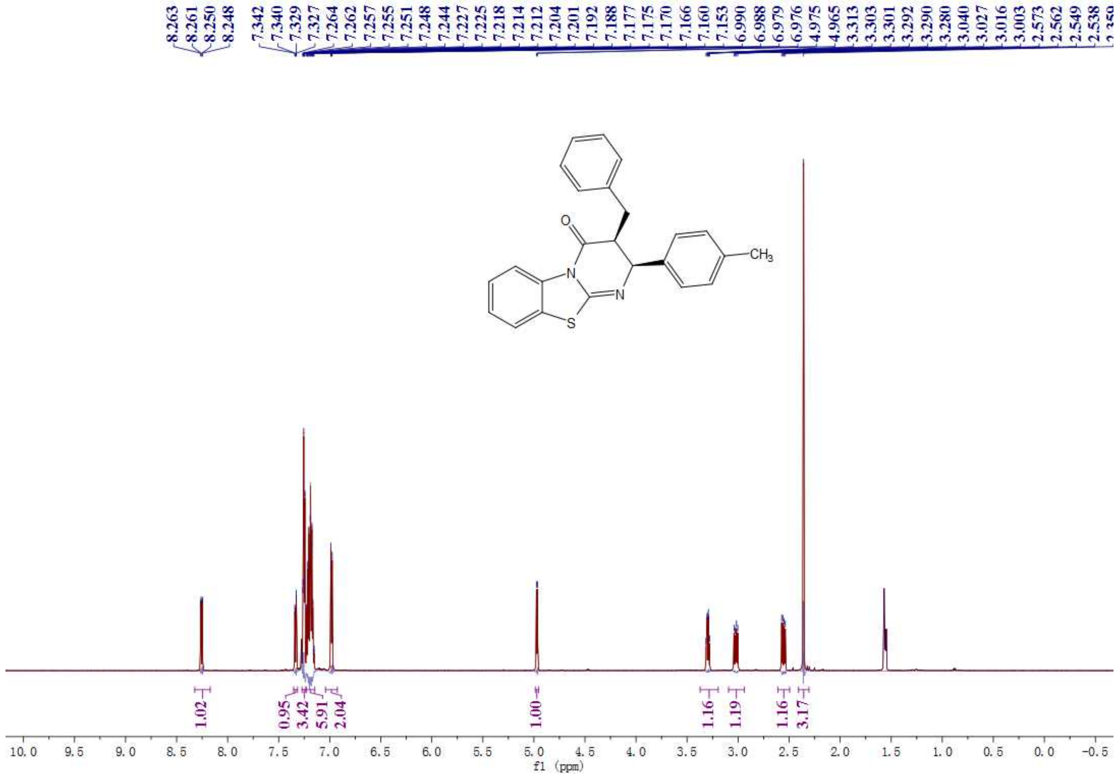

$^{13}$C NMR of **3b**

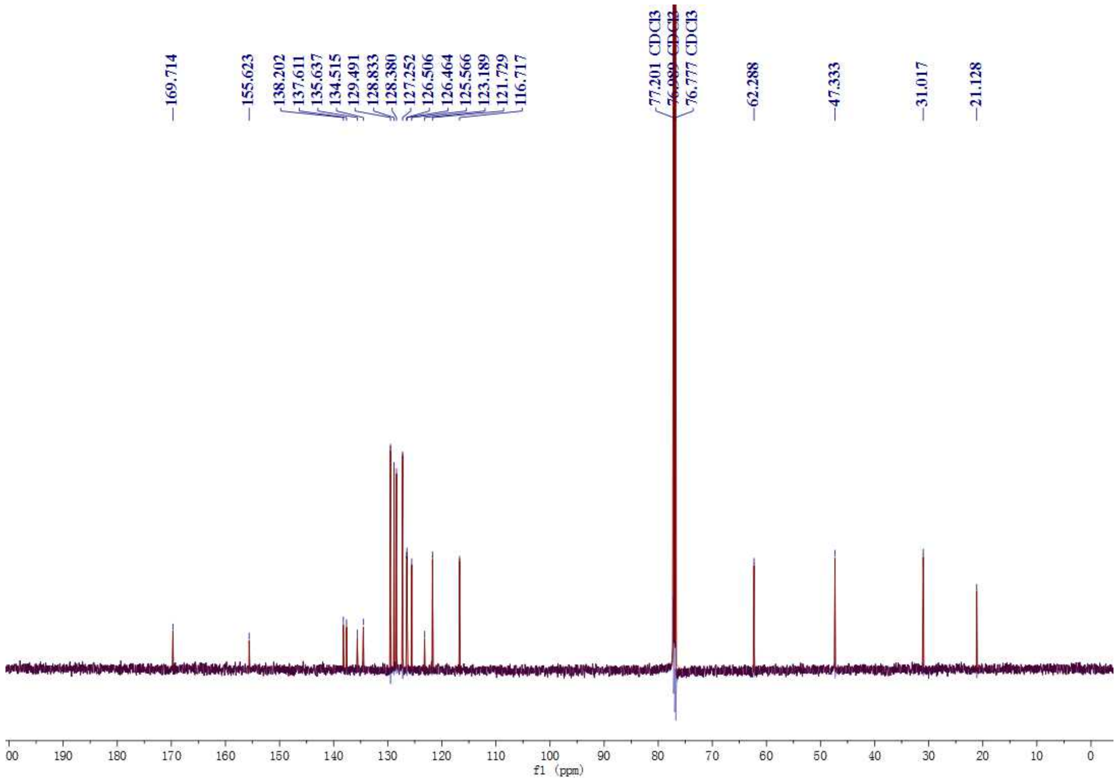

HPLC analysis: rac-**3b**

Sample name: **Ni 74 a**

Data file: C:\SNOOPY\NI\74AR1IA.D

Description: Laufmittel: n-Heptan/EtOH 7:3; Probe ist in LM/DCM gelöst.

Injection date: 8/22/2014 1:05:09 PM

Acq. Analysis method: CHIRALPAKIARNP.M

Column: Chiralpak IA, (250 x 4,6) mm, 5μ, SN: IA00CE-RC036

Pressure at start: 42 bar  Start flow: 0.700 ml/min  Column oven: 30 °C

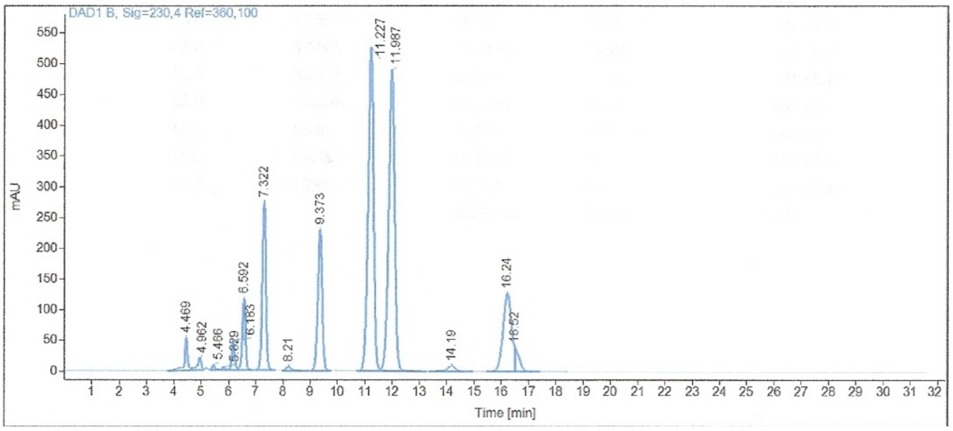


| RT [min] | Type | Area% | Area | Height | Width [min] |
|---|---|---|---|---|---|
| 4.47 | BV | 1.69 | 429.19 | 54.69 | 0.11 |
| 4.96 | VV | 0.78 | 199.02 | 20.47 | 0.14 |
| 5.47 | BB | 0.20 | 51.75 | 8.00 | 0.10 |
| 5.83 | BV | 0.12 | 29.65 | 4.45 | 0.11 |
| 6.18 | VV | 1.44 | 364.98 | 48.25 | 0.12 |
| 6.59 | VB | 3.59 | 911.82 | 117.22 | 0.12 |
| 7.32 | BV | 9.70 | 2463.09 | 273.82 | 0.14 |
| 8.21 | VB | 0.32 | 81.70 | 5.74 | 0.21 |
| 9.37 | BV | 10.41 | 2643.95 | 230.27 | 0.18 |
| 11.23 | BV | 28.93 | 7349.52 | 527.38 | 0.21 |
| 11.99 | VB | 29.37 | 7459.97 | 490.41 | 0.23 |
| 14.19 | BB | 0.78 | 197.50 | 9.30 | 0.31 |
| 16.24 | MF | 11.07 | 2811.93 | 127.40 | 0.37 |
| 16.52 | FM | 1.60 | 406.90 | 39.01 | 0.17 |
| | Sum | 100.00 | 25400.95 | | |

Enantioenriched **3b**

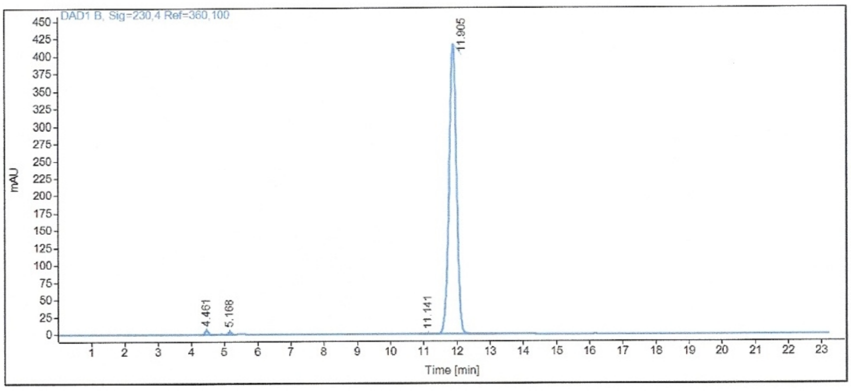


Name Ni 74 b

| RT [min] | Type | Area% | Area | Height | Width [min] |
|---|---|---|---|---|---|
| 4.46 | VV | 0.78 | 51.45 | 6.39 | 0.12 |
| 5.17 | VV | 0.48 | 31.42 | 4.21 | 0.11 |
| 11.14 | BB | 0.07 | 4.87 | 0.38 | 0.20 |
| 11.91 | BB | 98.67 | 6504.04 | 416.61 | 0.24 |
| | Sum | 100.00 | 6591.78 | | |

$^{1}$H NMR of **3c** (diastereomeric mixture)

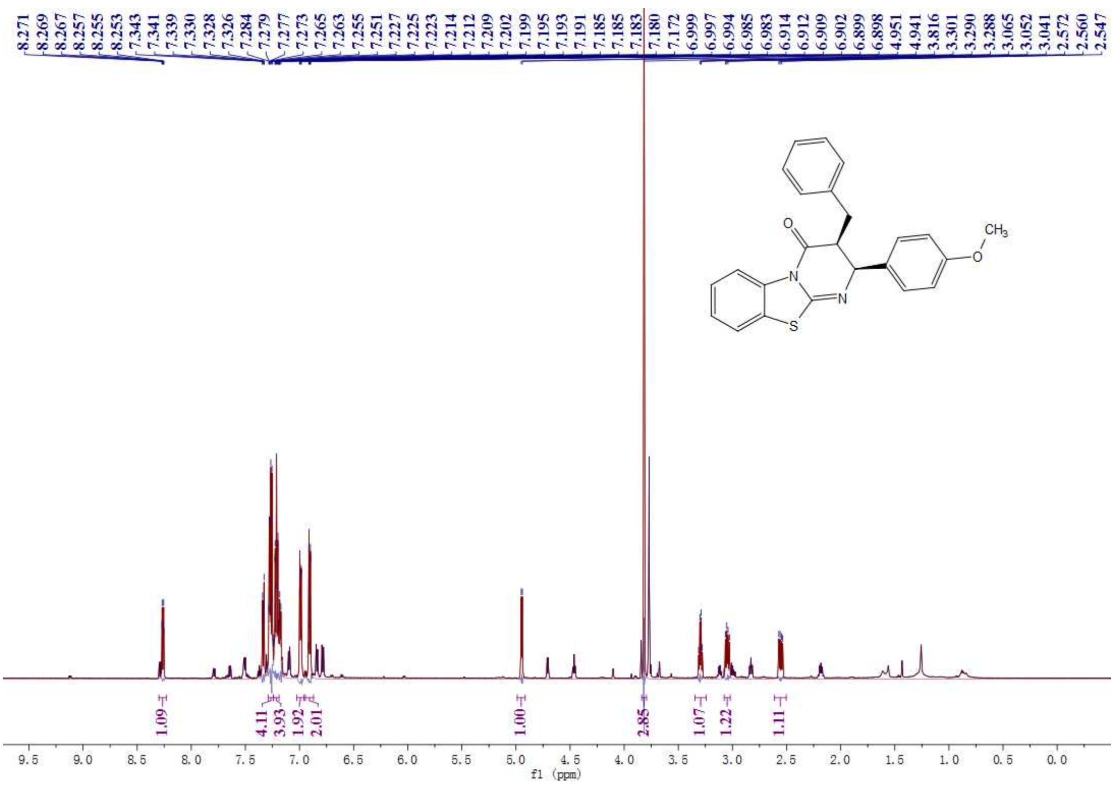


$^{13}$C NMR of **3c** (diastereomeric mixture)

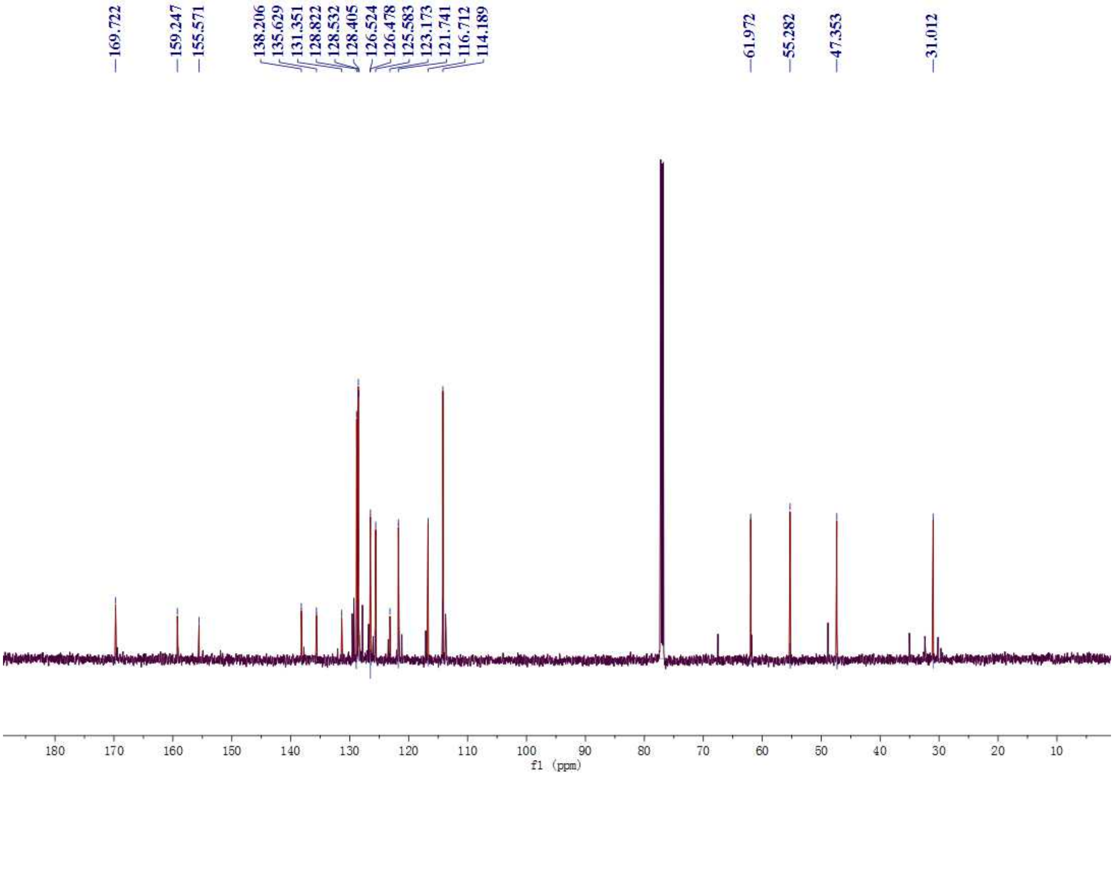

HPLC analysis: rac-**3c**

Sample name: **Ni 75 a rac**

Data file: C:\SNOOPY\NI\NI 75 A RAC IA.D

Description: Laufmittel: n-Heptan/EtOH 7:3 Die Probe ist DCM/LM gelöst.

Injection date: 8/28/2014 2:16:06 PM

Acq. Analysis method: CHIRALPAKIARNP.M

Column: Chiralpak IA, (250 x 4,6) mm, 5μ, SN: IA00CE-RC036

Pressure at start: 43 bar Start flow: 0.700 ml/min Column oven: 30 °C

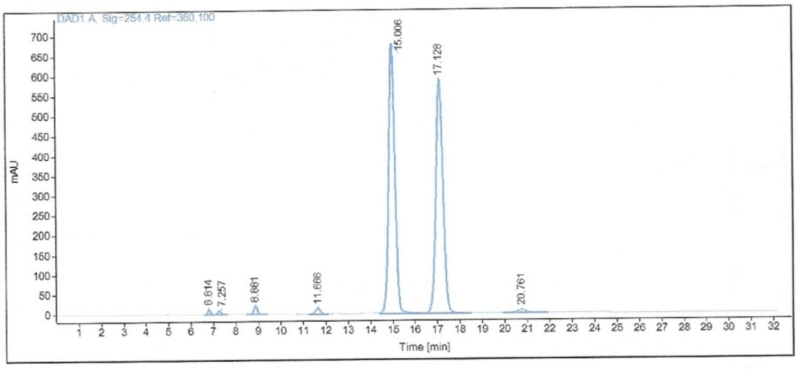


Name Ni 75 a rac

| RT [min] Type | Area% | Area | Height | Width [min] |
|---|---|---|---|---|
| 6.81 VV | 0.39 | 103.77 | 12.43 | 0.13 |
| 7.26 VV | 0.38 | 101.97 | 9.24 | 0.16 |
| 8.88 BB | 0.86 | 230.10 | 20.48 | 0.17 |
| 11.67 BV | 0.78 | 210.08 | 14.21 | 0.23 |
| 15.01 VB | 48.66 | 13052.96 | 678.87 | 0.30 |
| 17.13 BV | 48.15 | 12915.85 | 586.07 | 0.34 |
| 20.76 BB | 0.78 | 208.35 | 7.64 | 0.42 |
| Sum | 100.00 | 26823.08 | | |

Enantioenriched **3c**

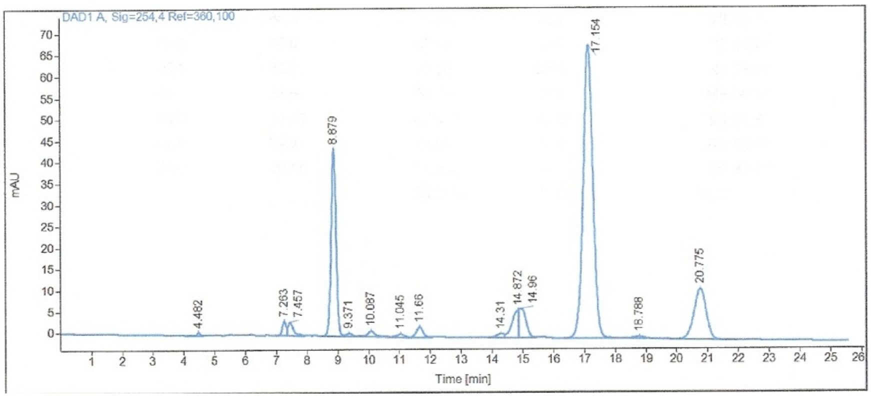


ame Ni 75 b

| RT [min] Type | Area% | Area | Height | Width [min] |
|---|---|---|---|---|
| 4.48 BV | 0.33 | 9.07 | 0.73 | 0.17 |
| 7.26 BV | 1.09 | 29.71 | 3.29 | 0.14 |
| 7.46 VV | 1.49 | 40.63 | 3.16 | 0.19 |
| 8.88 BV | 17.80 | 486.18 | 43.81 | 0.17 |
| 9.37 VV | 0.36 | 9.70 | 0.74 | 0.20 |
| 10.09 VV | 0.92 | 25.03 | 1.33 | 0.27 |
| 11.05 VB | 0.41 | 11.24 | 0.68 | 0.25 |
| 11.66 BV | 1.29 | 35.23 | 2.36 | 0.23 |
| 14.31 BV | 0.57 | 15.63 | 0.91 | 0.26 |
| 14.87 MF | 3.52 | 96.04 | 6.55 | 0.24 |
| 14.96 FM | 3.95 | 107.99 | 6.86 | 0.26 |
| 17.15 BB | 55.84 | 1525.20 | 68.60 | 0.34 |
| 18.79 BB | 0.51 | 13.80 | 0.46 | 0.43 |
| 20.78 BB | 11.93 | 325.77 | 11.93 | 0.43 |
| Sum | 100.00 | 2731.22 | | |

$^{1}$H NMR of **3d**

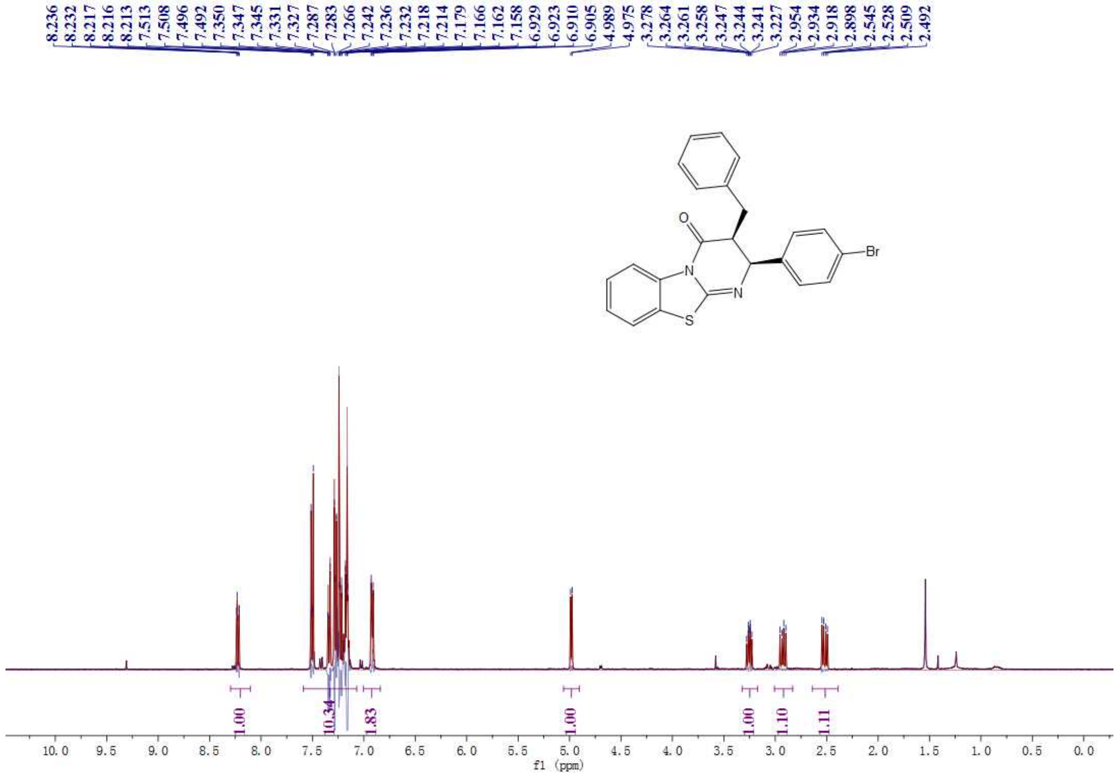

$^{13}$C NMR of **3d**

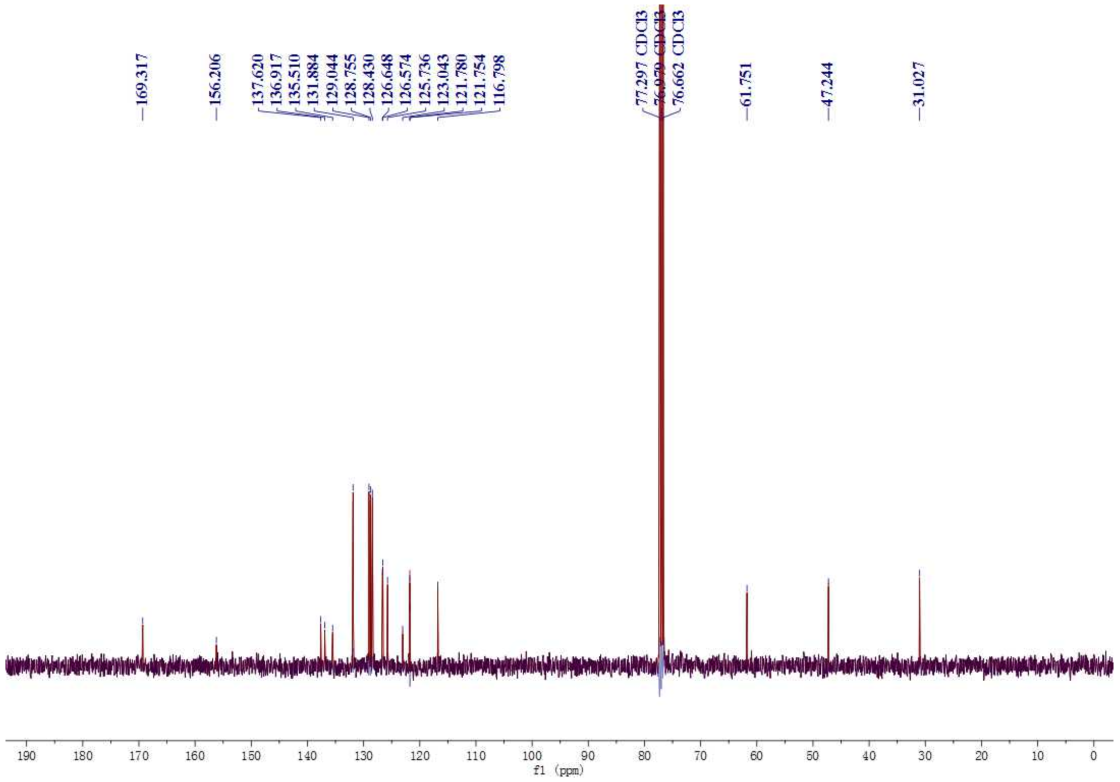

HPLC analysis: rac-**3d**

**Sample name:** Ni 76 a rac

**Data file:** C:\SNOOPY\NI\NI 76 A RAC IA.D
**Description:** Laufmittel: n-Heptan/EtOH 7:3 Die Probe ist DCM/LM gelöst.

**Injection date:** 8/14/2014 2:29:12 PM
**Acq. Analysis method:** CHIRALPAKIARNP.M

**Column:** Chiralpak IA, (250 x 4,6) mm, 5μ, SN: IA00CE-RC036

**Pressure at start:** 42 bar **Start flow:** 0.700 ml/min **Column oven:** 29.99 °C

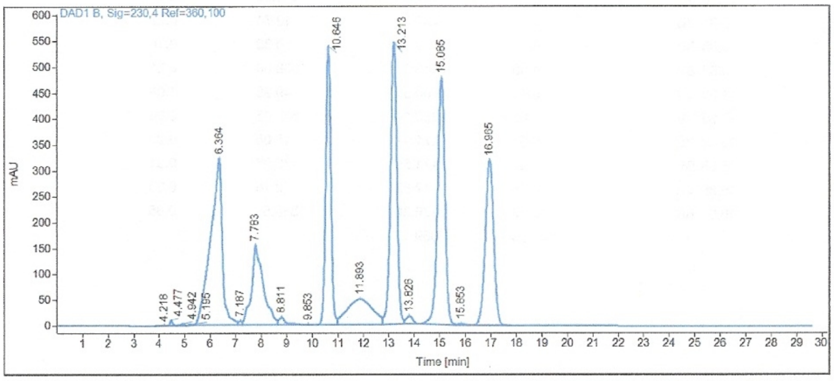


| RT [min] | Type | Area% | Area | Height | Width [min] |
|---|---|---|---|---|---|
| 4.22 | BV | 0.02 | 9.85 | 0.85 | 0.18 |
| 4.48 | VV | 0.15 | 80.04 | 10.98 | 0.11 |
| 4.94 | VB | 0.08 | 40.60 | 4.26 | 0.13 |
| 5.19 | BB | 0.03 | 15.47 | 2.78 | 0.09 |
| 6.36 | BV | 19.54 | 10359.40 | 322.88 | 0.41 |
| 7.19 | VV | 0.14 | 71.67 | 7.01 | 0.15 |
| 7.78 | VV | 9.27 | 4911.40 | 154.02 | 0.41 |
| 8.81 | VB | 0.51 | 267.86 | 13.74 | 0.27 |
| 9.85 | BB | 0.01 | 3.58 | 0.33 | 0.18 |
| 10.65 | BV | 14.05 | 7448.01 | 538.86 | 0.21 |
| 11.89 | VV | 6.41 | 3400.31 | 49.28 | 1.08 |
| 13.21 | VV | 17.62 | 9339.14 | 545.05 | 0.26 |
| 13.83 | VB | 0.51 | 271.11 | 15.05 | 0.27 |
| 15.08 | BV | 17.87 | 9471.59 | 476.67 | 0.31 |
| 15.85 | VB | 0.08 | 42.82 | 2.16 | 0.30 |
| 16.97 | BB | 13.73 | 7276.30 | 318.59 | 0.35 |
| | Sum | 100.00 | 53009.15 | | |

Enantioenriched **3d**

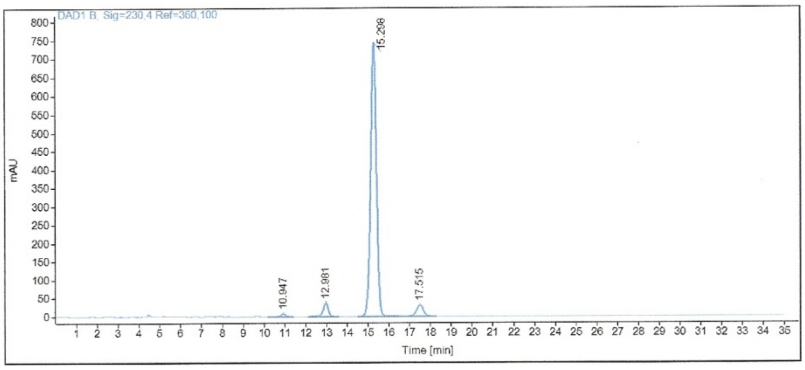


**Name** Ni 76 b

| RT [min] | Type | Area% | Area | Height | Width [min] |
|---|---|---|---|---|---|
| 10.95 | BB | 0.67 | 106.96 | 7.62 | 0.22 |
| 12.98 | BB | 4.05 | 645.84 | 36.73 | 0.27 |
| 15.30 | BB | 90.93 | 14487.28 | 744.18 | 0.30 |
| 17.51 | BB | 4.34 | 691.59 | 30.10 | 0.35 |
| | Sum | 100.00 | 15931.67 | | |

$^{1}$H NMR of **3e**

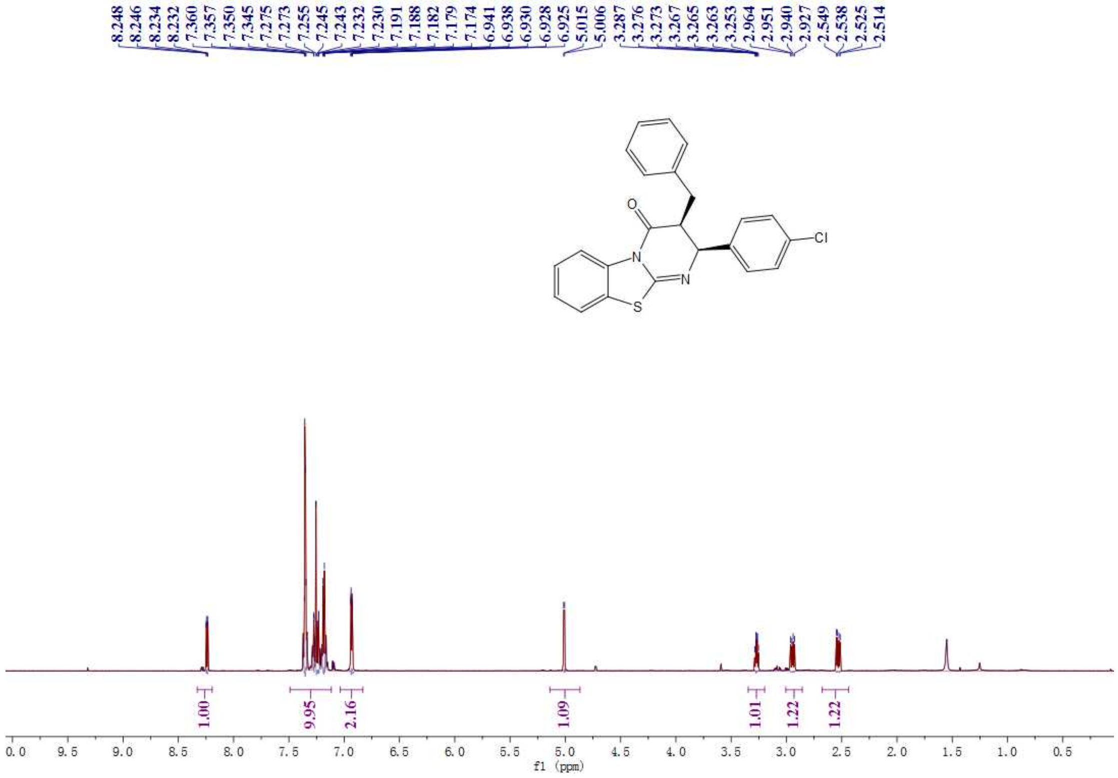


$^{13}$C NMR of **3e**

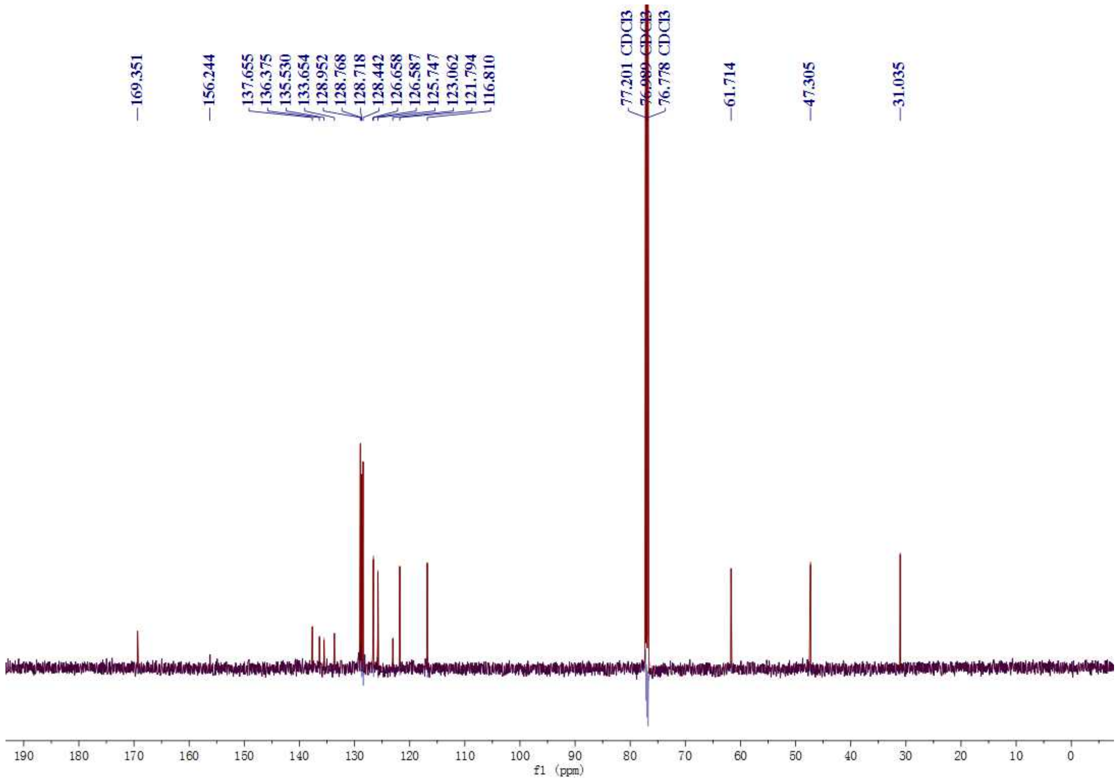

HPLC analysis: rac-**3e**

HEWLETT PACKARD

| | |
|---|---|
| Sample Name: | Ni 77 a rac |
| Data file: | D:\GONZO\NI\77ARAD.D |
| Sample Info: | Laufmittel: n-Heptan/~~IP~~ EtOH 9:1; |
| | Die Probe ist in DCM/LM gelöst. |
| Säule: | DAICELAD.M |
| Säuleninfo: | Chiralpak AD (250x4,6)mm |
| Operator: | Analytik Labor AKEN |
| Injektion Time: | 10:48:37 |
| Injektion Date: | 21.08.2014 |

| Instrument Conditions: | At Start | At Stop |
|---|---|---|
| Temperature in°C: | 30.0°C | 30.0°C |
| Pressure in bar: | 25.8 | 26.3 |
| Flow in ml/min: | 1.00 | 1.00 |

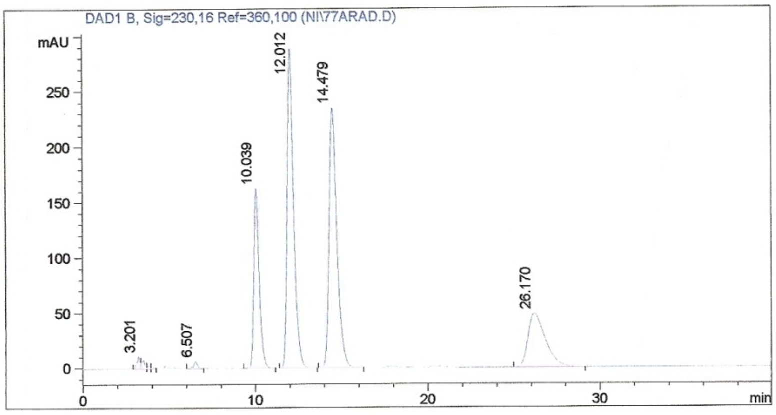


| # | Ret. Time (min) | Width | Height (mAU) | Area (mAU*s) | Area % |
|---|---|---|---|---|---|
| 1 | 3.20 | 0.18 | 10.98 | 141.85 | 0.64 |
| 2 | 3.47 | 0.17 | 8.20 | 103.18 | 0.46 |
| 3 | 3.80 | 0.15 | 3.21 | 30.98 | 0.14 |
| 4 | 4.00 | 0.14 | 1.98 | 19.57 | 0.09 |
| 5 | 6.51 | 0.24 | 6.59 | 112.04 | 0.50 |
| 6 | 10.04 | 0.32 | 162.47 | 3519.73 | 15.79 |
| 7 | 12.01 | 0.39 | 288.53 | 7475.61 | 33.54 |
| 8 | 14.48 | 0.48 | 234.62 | 7422.56 | 33.30 |
| 9 | 26.17 | 1.09 | 48.56 | 3461.63 | 15.53 |
| Total | | | | 22287.13 | 100.00 |

Enantioenriched **3e**

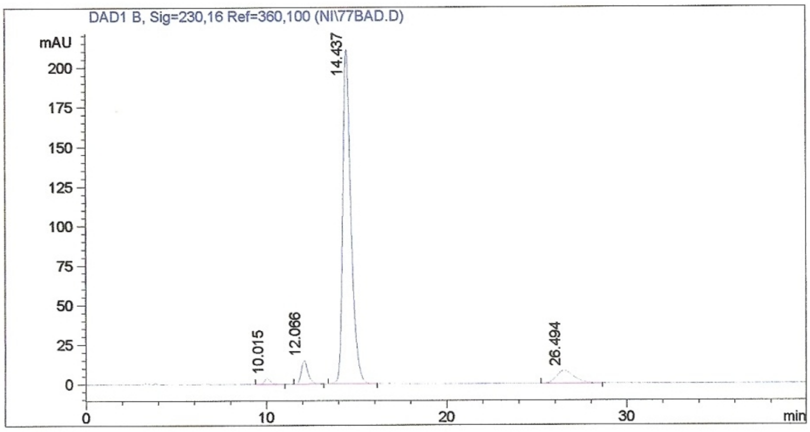


| # | Ret. Time (min) | Width | Height (mAU) | Area (mAU*s) | Area % |
|---|---|---|---|---|---|
| 1 | 10.01 | 0.34 | 3.68 | 84.05 | 1.09 |
| 2 | 12.07 | 0.39 | 14.90 | 386.22 | 4.99 |
| 3 | 14.44 | 0.48 | 211.29 | 6714.22 | 86.79 |
| 4 | 26.49 | 1.05 | 8.07 | 551.93 | 7.13 |
| Total | | | | 7736.42 | 100.00 |

$^{1}H$ NMR of **3f**

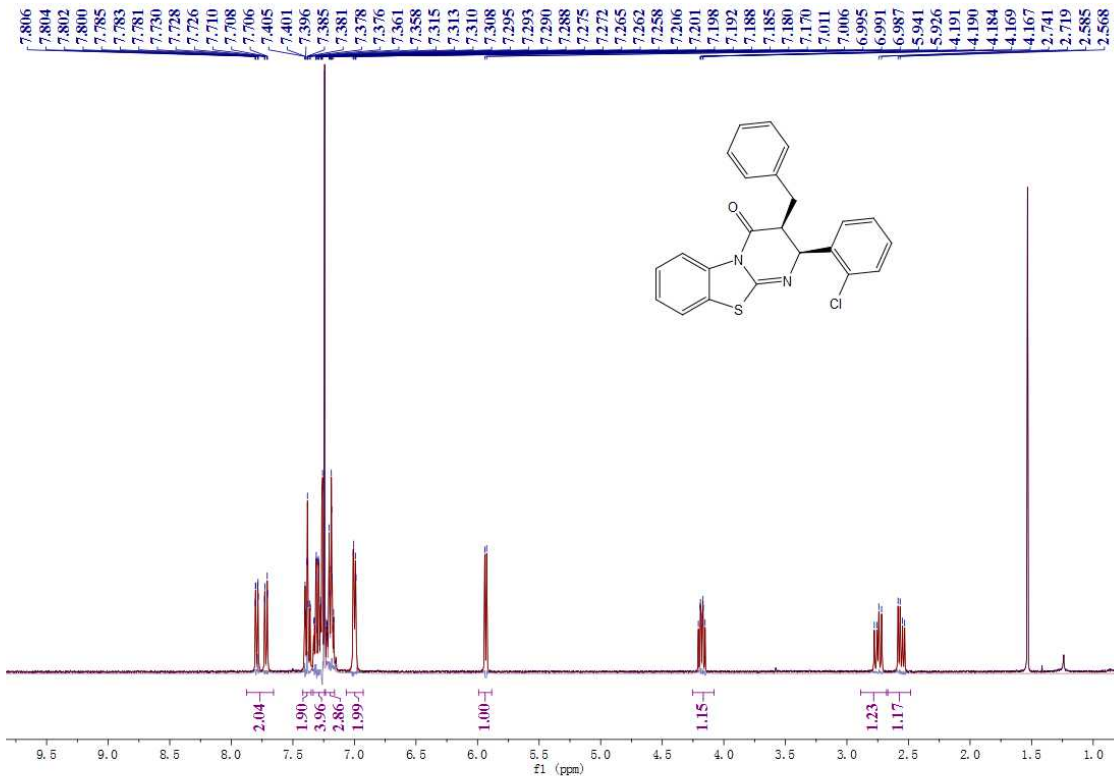


$^{13}C$ NMR of **3f**

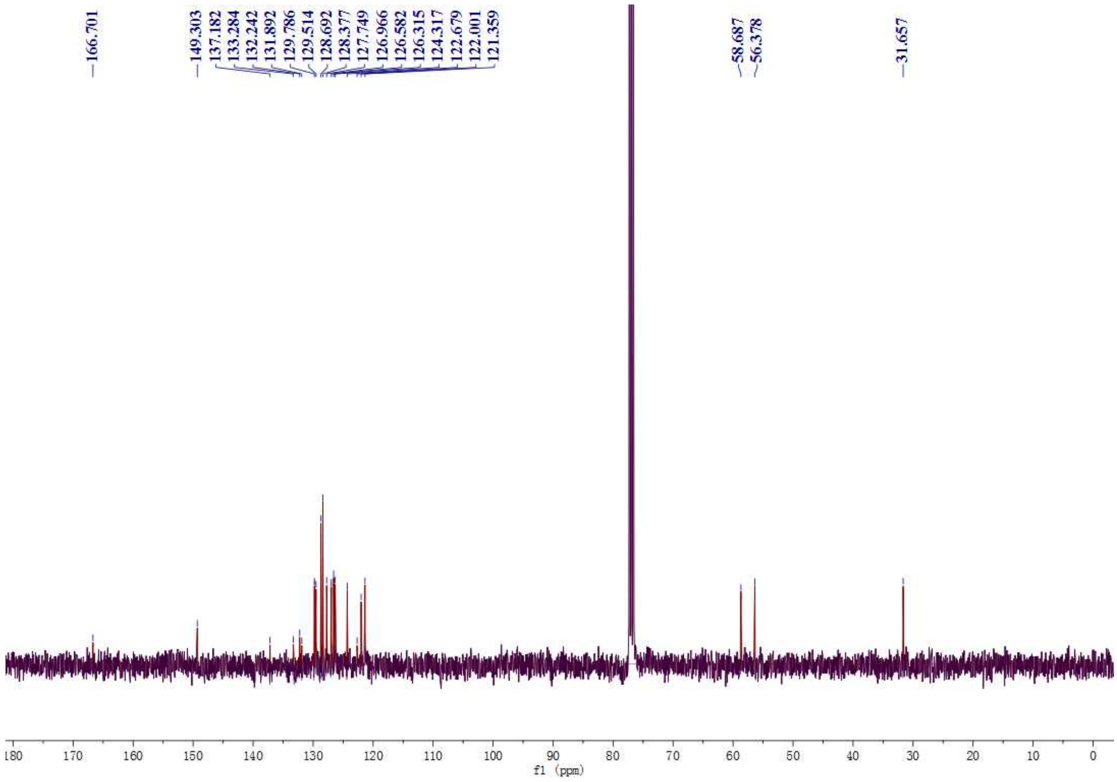

HPLC analysis: rac-**3f**

Sample Name: Ni 85 a neu
Data file: D:\BERT\NI\85ANOJ.D
Sample Info: Laufmittel: n-Heptan/EtOH 7:3;
Die Probe ist in DCM/LM gelöst

Agilent Technologies

Säule: DAICELOJ.M
Säuleninfo: Chiralcel OJ (250x4,6)mm

Operator: Analytik Labor AKEN

Injektion Time: 09:58:41
Injektion Date: 17.09.2014

| Instrument Conditions: | At Start | At Stop |
|---|---|---|
| Temperature in°C: | 30.0 | 30.0 |
| Pressure in bar: | 33.5 | 33.1 |
| Flow in ml/min: | 0.7 | 0.7 |

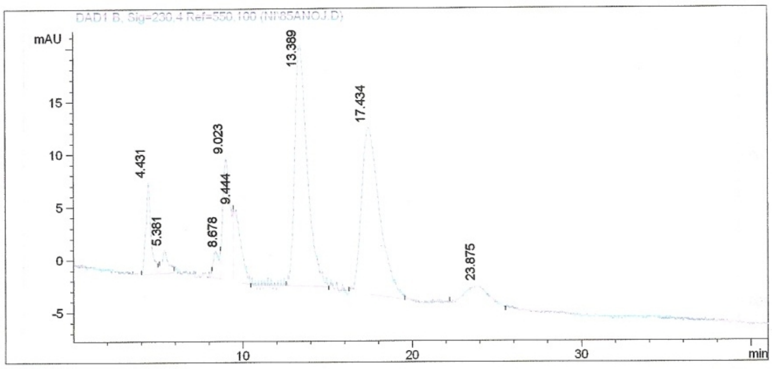


| # | Ret. Time (min) | Width | Height (mAU) | Area (mAU*s) | Area % |
|---|---|---|---|---|---|
| 1 | 4.43 | 0.30 | 8.76 | 189.27 | 5.59 |
| 2 | 5.38 | 0.36 | 2.27 | 59.68 | 1.76 |
| 3 | 8.68 | 0.40 | 2.65 | 63.18 | 1.87 |
| 4 | 9.02 | 0.52 | 11.58 | 361.97 | 10.69 |
| 5 | 9.44 | 0.49 | 6.95 | 204.42 | 6.04 |
| 6 | 13.39 | 0.86 | 22.85 | 1174.67 | 34.69 |
| 7 | 17.43 | 0.99 | 15.88 | 1176.42 | 34.74 |
| 8 | 23.87 | 1.43 | 1.82 | 156.94 | 4.63 |
| Total | | | | 3386.55 | 100.00 |

Enantioenriched **3f**

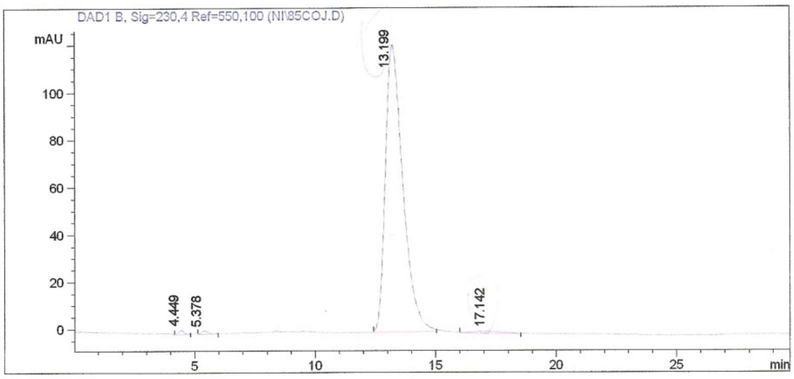


| # | Ret. Time (min) | Width | Height (mAU) | Area (mAU*s) | Area % |
|---|---|---|---|---|---|
| 1 | 4.45 | 0.24 | 1.81 | 31.54 | 0.50 |
| 2 | 5.38 | 0.27 | 1.30 | 24.29 | 0.39 |
| 3 | 13.20 | 0.75 | 121.34 | 6140.56 | 97.85 |
| 4 | 17.14 | 1.31 | 1.00 | 78.95 | 1.26 |
| Total | | | | 6275.34 | 100.00 |

$^{1}$H NMR of **3g**

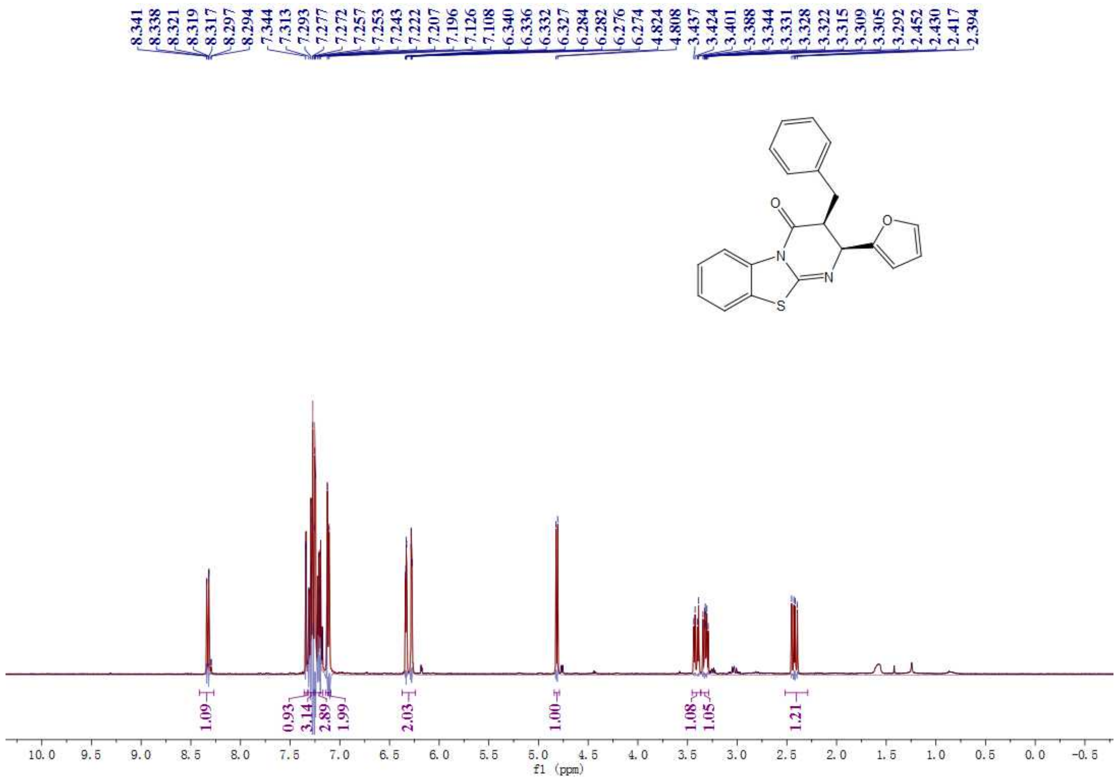


$^{13}$C NMR of **3g**

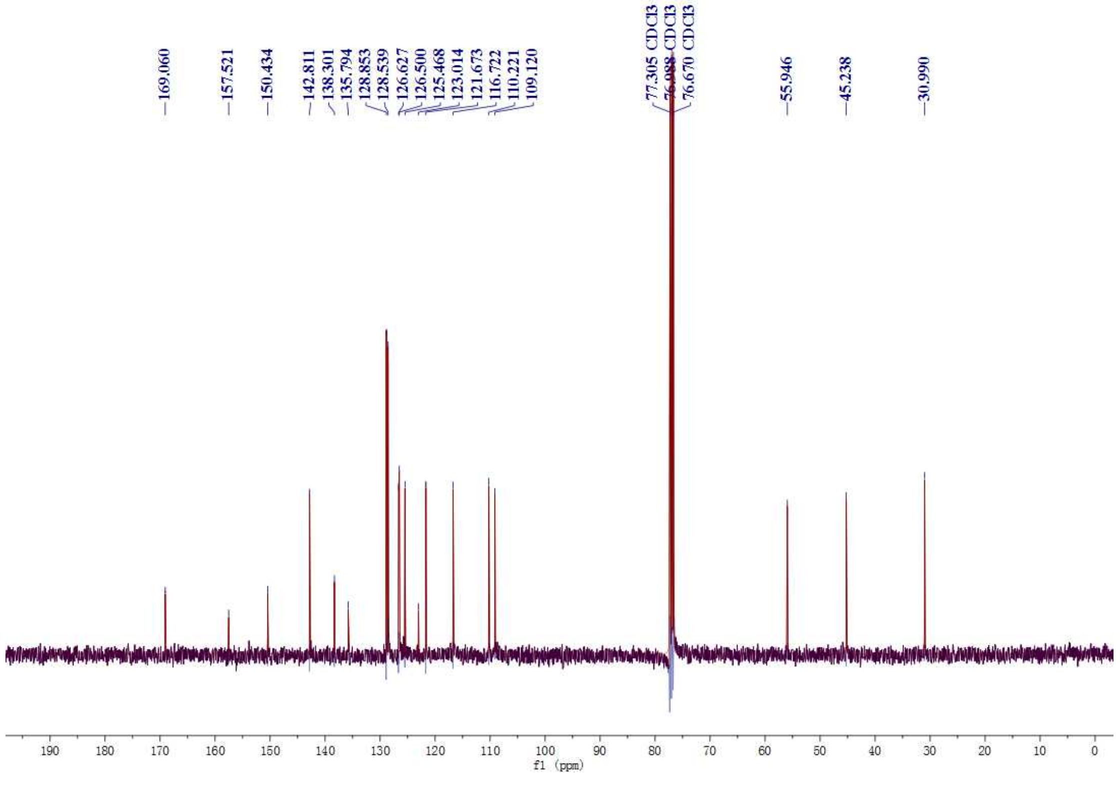

HPLC analysis: rac-**3g**

HEWLETT PACKARD

| | | | |
|---|---|---|---|
| Sample Name: | Ni 82 a rac | | |
| Data file: | D:\GONZO\NI\82ARAD.D | | |
| Sample Info: | Laufmittel: n-Heptan/EtOH 7:3 | | |
| | Die Probe ist in LM/DCM gelöst | | |
| Säule: | DAICELAD.M | | |
| Säuleninfo: | Chiralpak AD (250x4,6)mm | | |
| Operator: | Analytik Labor AKEN | | |
| Injektion Time: | 19:18:56 | | |
| Injektion Date: | 25.08.2014 | | |
| Instrument Conditions: | At Start | At Stop | |
| Temperature in°C: | 30.0°C | 30.0°C | |
| Pressure in bar: | 22.1 | 22.5 | |
| Flow in ml/min: | 0.70 | 0.70 | |

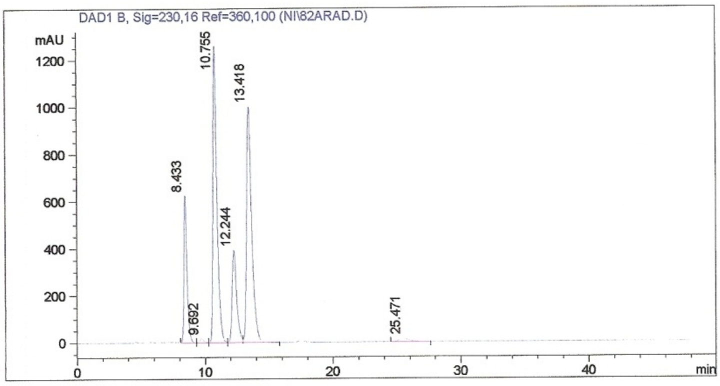


| # | Ret. Time (min) | Width | Height (mAU) | Area (mAU*s) | Area % |
|---|---|---|---|---|---|
| 1 | 8.43 | 0.24 | 625.92 | 10228.92 | 13.42 |
| 2 | 9.69 | 0.33 | 17.14 | 397.80 | 0.52 |
| 3 | 10.75 | 0.32 | 1260.34 | 27133.10 | 35.59 |
| 4 | 12.24 | 0.37 | 392.53 | 9759.25 | 12.80 |
| 5 | 13.42 | 0.42 | 1001.63 | 27954.02 | 36.67 |
| 6 | 25.47 | 0.77 | 14.87 | 764.42 | 1.00 |
| Total | | | | 76237.51 | 100.00 |

Enantioenriched **3g**

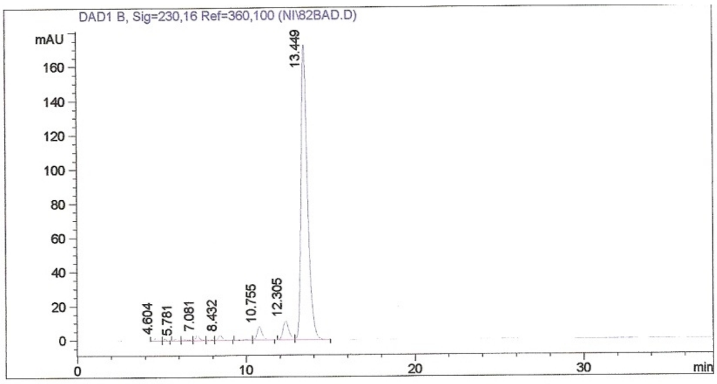


| # | Ret. Time (min) | Width | Height (mAU) | Area (mAU*s) | Area % |
|---|---|---|---|---|---|
| 1 | 4.60 | 0.22 | 1.55 | 25.17 | 0.48 |
| 2 | 5.15 | 0.16 | 1.27 | 13.81 | 0.26 |
| 3 | 5.78 | 0.22 | 0.99 | 15.59 | 0.30 |
| 4 | 6.31 | 0.32 | 0.60 | 14.58 | 0.28 |
| 5 | 7.08 | 0.22 | 3.38 | 50.83 | 0.96 |
| 6 | 7.95 | 0.35 | 0.27 | 7.09 | 0.13 |
| 7 | 8.43 | 0.27 | 3.12 | 56.33 | 1.07 |
| 8 | 10.00 | 0.52 | 0.47 | 18.65 | 0.35 |
| 9 | 10.75 | 0.32 | 7.96 | 167.92 | 3.19 |
| 10 | 12.30 | 0.37 | 11.38 | 273.58 | 5.19 |
| 11 | 13.45 | 0.41 | 172.62 | 4626.01 | 87.79 |
| Total | | | | 5269.56 | 100.00 |

$^{1}$H NMR of **3h**

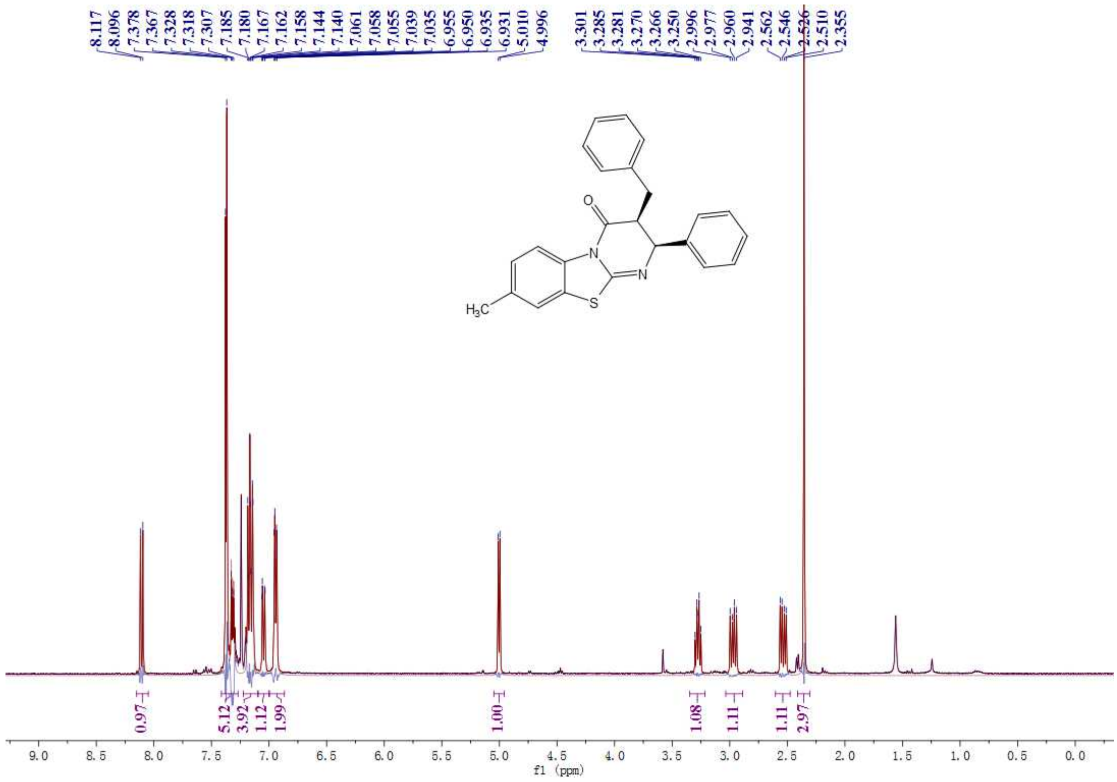


$^{13}$C NMR of **3h**

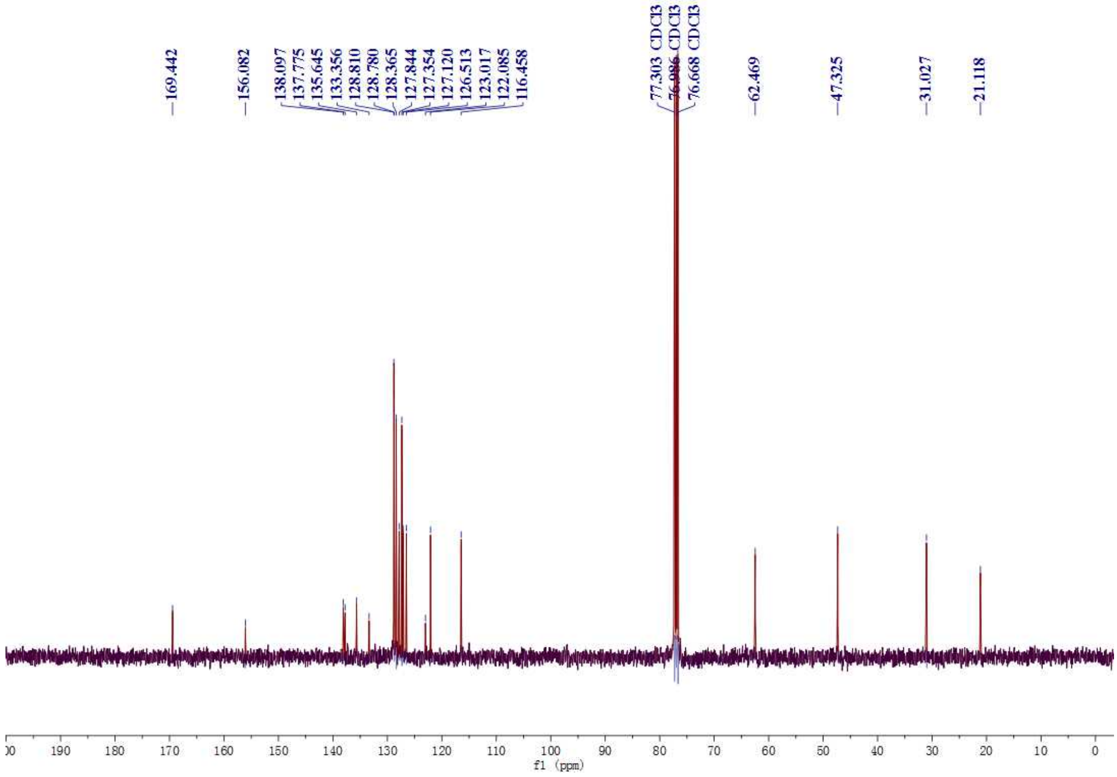

HPLC analysis: rac-**3h**

Sample name: **Ni 80 a rac**

Data file: C:\SNOOPY\NI\NI 80 A RAC 1IC.D

Description: Laufmittel: n-Heptan/IP 9:1 Die Probe ist DCM/LM gelöst.

Injection date: 8/25/2014 2:05:07 PM

Acq. Analysis method: CHIRALPAKIC1-6LNP.M

Column: Chiralpak IC, (150 x 4,6) mm, 5µ, SN: IC00CD-QF015

Pressure at start: 22 bar Start flow: 0.500 ml/min Column oven: 30 °C

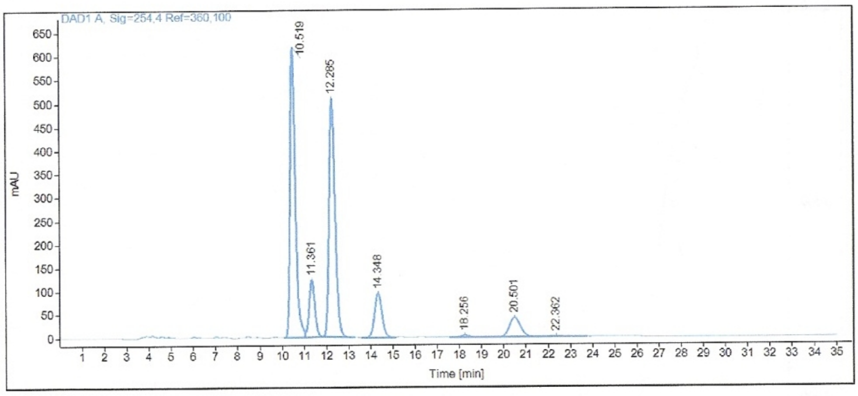


Name Ni 80 a rac

| RT [min] | Type | Area% | Area | Height | Width [min] |
|---|---|---|---|---|---|
| 10.52 | BV | 39.27 | 10178.03 | 618.48 | 0.25 |
| 11.36 | VB | 8.43 | 2185.63 | 122.66 | 0.28 |
| 12.29 | BB | 37.32 | 9672.71 | 508.46 | 0.29 |
| 14.35 | BV | 8.45 | 2190.20 | 94.89 | 0.36 |
| 18.26 | BB | 0.50 | 128.51 | 4.12 | 0.48 |
| 20.50 | BV | 5.67 | 1468.64 | 41.65 | 0.54 |
| 22.36 | VB | 0.36 | 93.18 | 2.37 | 0.60 |
| | Sum | 100.00 | 25916.89 | | |

Enantioenriched **3h**

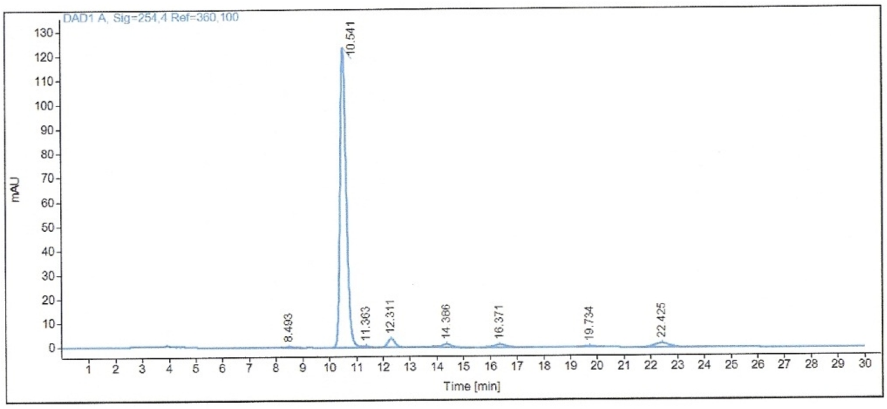


Name Ni 80 b

| RT [min] | Type | Area% | Area | Height | Width [min] |
|---|---|---|---|---|---|
| 8.49 | BB | 0.25 | 5.70 | 0.45 | 0.19 |
| 10.54 | BV | 89.02 | 1991.12 | 123.65 | 0.25 |
| 11.36 | VV | 0.51 | 11.40 | 0.56 | 0.30 |
| 12.31 | VB | 3.26 | 72.95 | 3.62 | 0.31 |
| 14.39 | BB | 1.74 | 38.82 | 1.34 | 0.42 |
| 16.37 | BB | 1.75 | 39.10 | 1.21 | 0.47 |
| 19.73 | BB | 0.56 | 12.47 | 0.46 | 0.43 |
| 22.43 | BB | 2.91 | 65.17 | 1.66 | 0.60 |
| | Sum | 100.00 | 2236.74 | | |

$^{1}$H NMR of **3i**

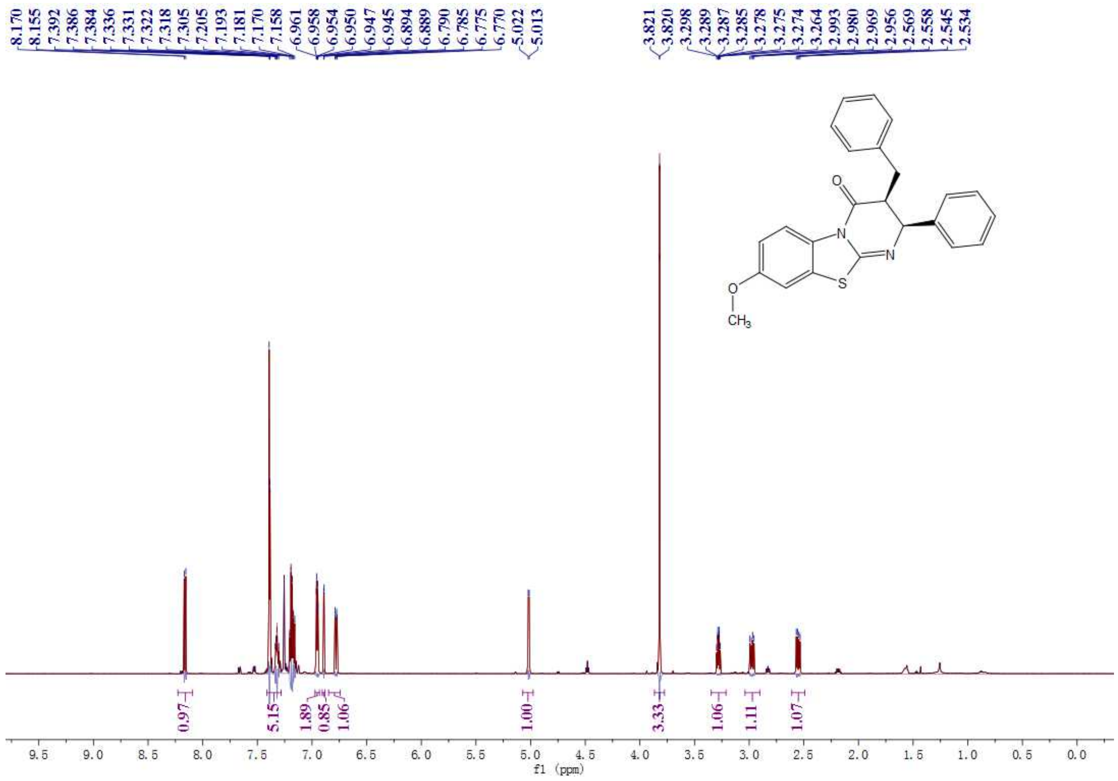


$^{13}$C NMR of **3i**

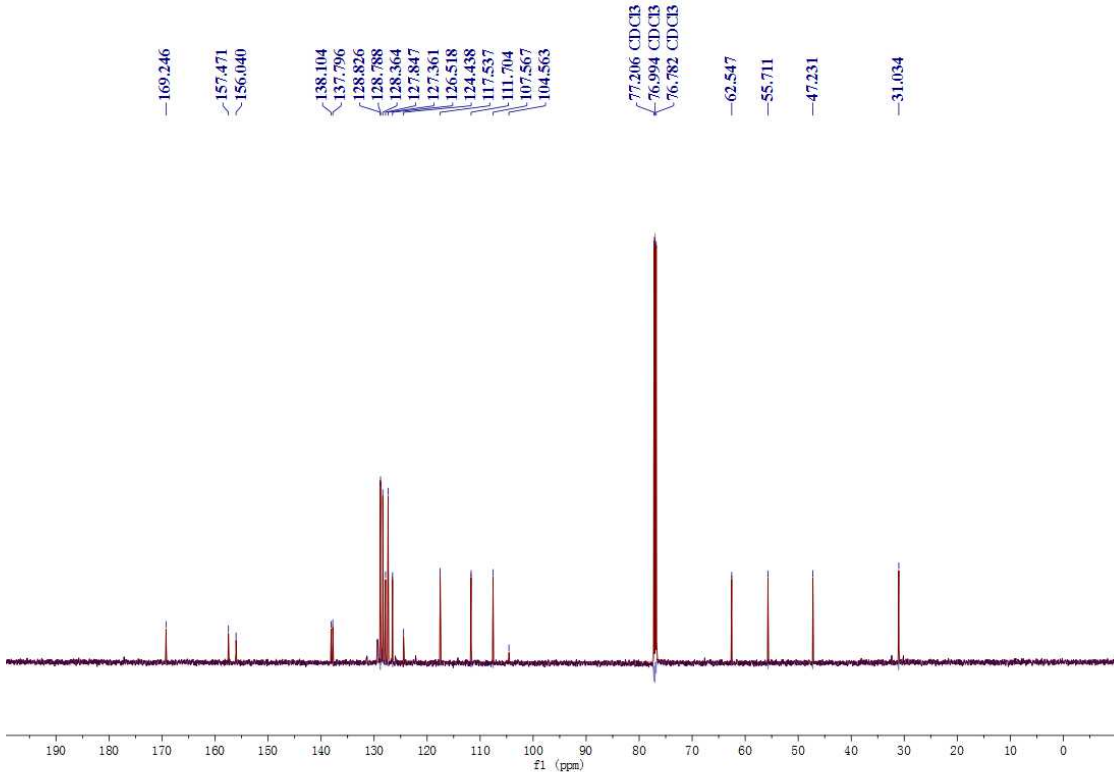

HPLC analysis: rac-**3i**

Sample Name: Ni 81 a rac
Data file: D:\GONZO\NI\81ARAD.D
Sample Info: Laufmittel: n-Heptan/EtOH 7:3
Die Probe ist in LM/DCM gelöst

Säule: DAICELAD.M
Säuleninfo: Chiralpak AD (250x4,6)mm
Operator: Analytik Labor AKEN

Injektion Time: 18:27:45
Injektion Date: 25.08.2014

| Instrument Conditions: | At Start | At Stop |
|---|---|---|
| Temperature in°C: | 30.0°C | 30.0°C |
| Pressure in bar: | 21.9 | 22.9 |
| Flow in ml/min: | 0.70 | 0.70 |

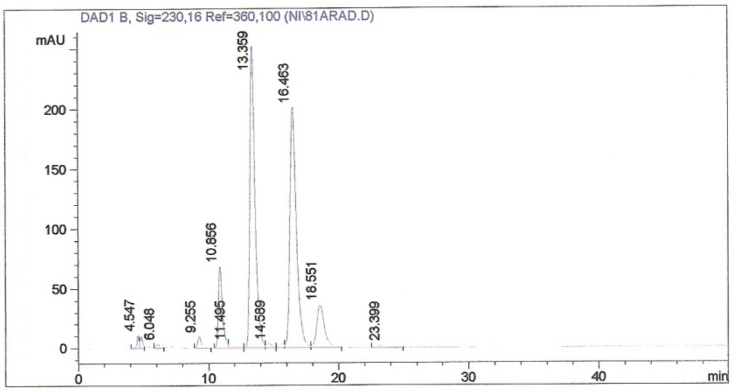


| # | Ret. Time (min) | Width | Height (mAU) | Area (mAU*s) | Area % |
|---|---|---|---|---|---|
| 1 | 4.55 | 0.16 | 11.16 | 123.89 | 0.69 |
| 2 | 4.80 | 0.19 | 10.05 | 128.44 | 0.71 |
| 3 | 6.05 | 0.29 | 3.87 | 77.36 | 0.43 |
| 4 | 9.26 | 0.30 | 9.70 | 193.23 | 1.07 |
| 5 | 10.86 | 0.37 | 68.54 | 1540.84 | 8.57 |
| 6 | 11.50 | 0.46 | 3.66 | 100.57 | 0.56 |
| 7 | 13.36 | 0.41 | 252.27 | 6948.46 | 38.63 |
| 8 | 14.59 | 0.44 | 3.96 | 116.86 | 0.65 |
| 9 | 15.64 | 0.38 | 3.62 | 89.35 | 0.50 |
| 10 | 16.46 | 0.53 | 201.23 | 7081.27 | 39.37 |
| 11 | 18.55 | 0.61 | 36.66 | 1473.66 | 8.19 |
| 12 | 23.40 | 0.77 | 2.11 | 111.49 | 0.62 |
| Total | | | | 17985.41 | 100.00 |

Enantioenriched **3i**

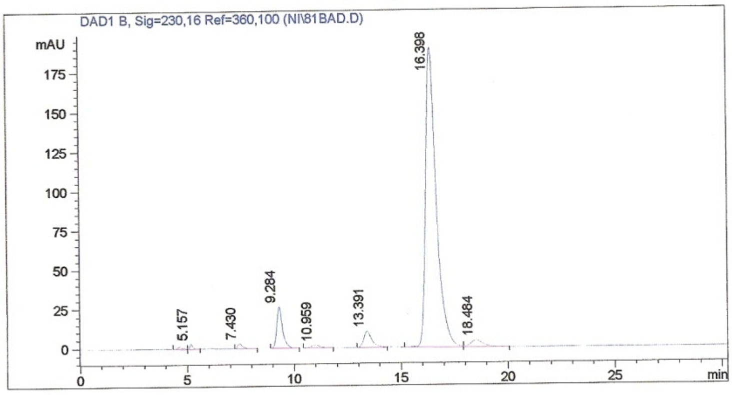


| # | Ret. Time (min) | Width | Height (mAU) | Area (mAU*s) | Area % |
|---|---|---|---|---|---|
| 1 | 4.61 | 0.25 | 1.53 | 28.73 | 0.37 |
| 2 | 5.16 | 0.16 | 3.32 | 36.66 | 0.48 |
| 3 | 7.43 | 0.22 | 3.22 | 47.76 | 0.62 |
| 4 | 9.28 | 0.28 | 26.35 | 495.07 | 6.41 |
| 5 | 10.96 | 0.49 | 1.64 | 50.74 | 0.66 |
| 6 | 13.39 | 0.41 | 10.39 | 284.26 | 3.68 |
| 7 | 16.40 | 0.52 | 190.29 | 6591.13 | 85.40 |
| 8 | 18.48 | 0.60 | 4.57 | 183.66 | 2.38 |
| Total | | | | 7718.02 | 100.00 |

$^{1}$H NMR of **3j**

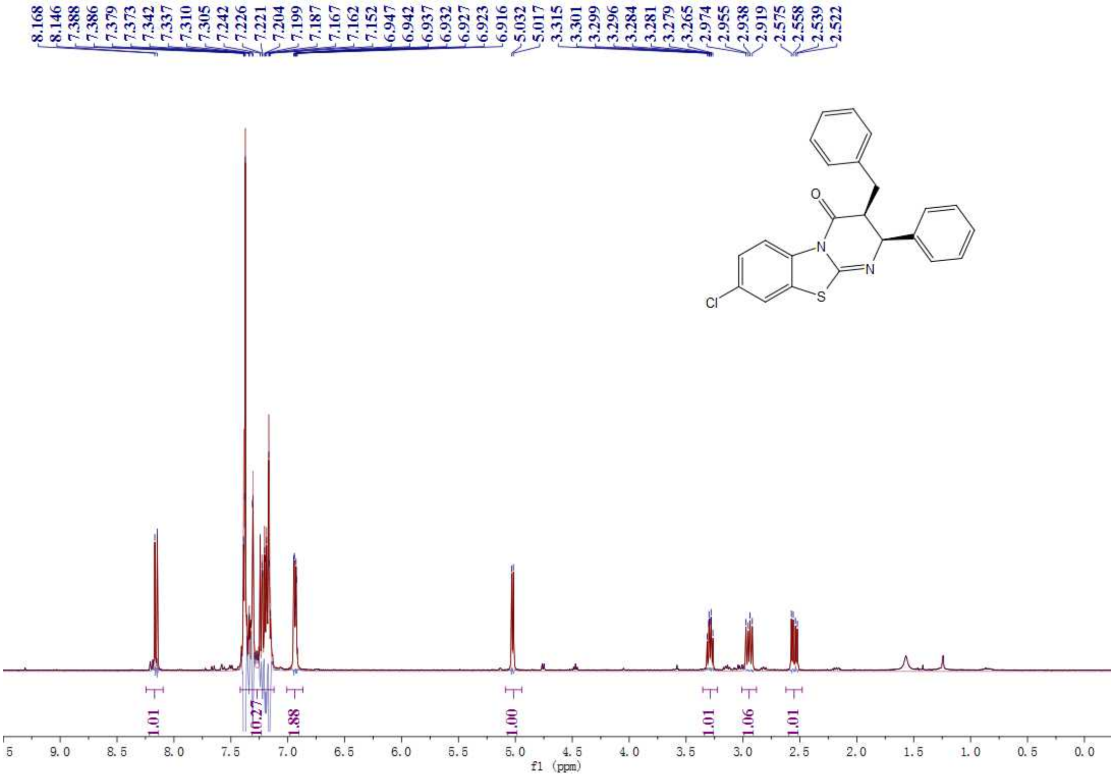


$^{13}$C NMR of **3j**

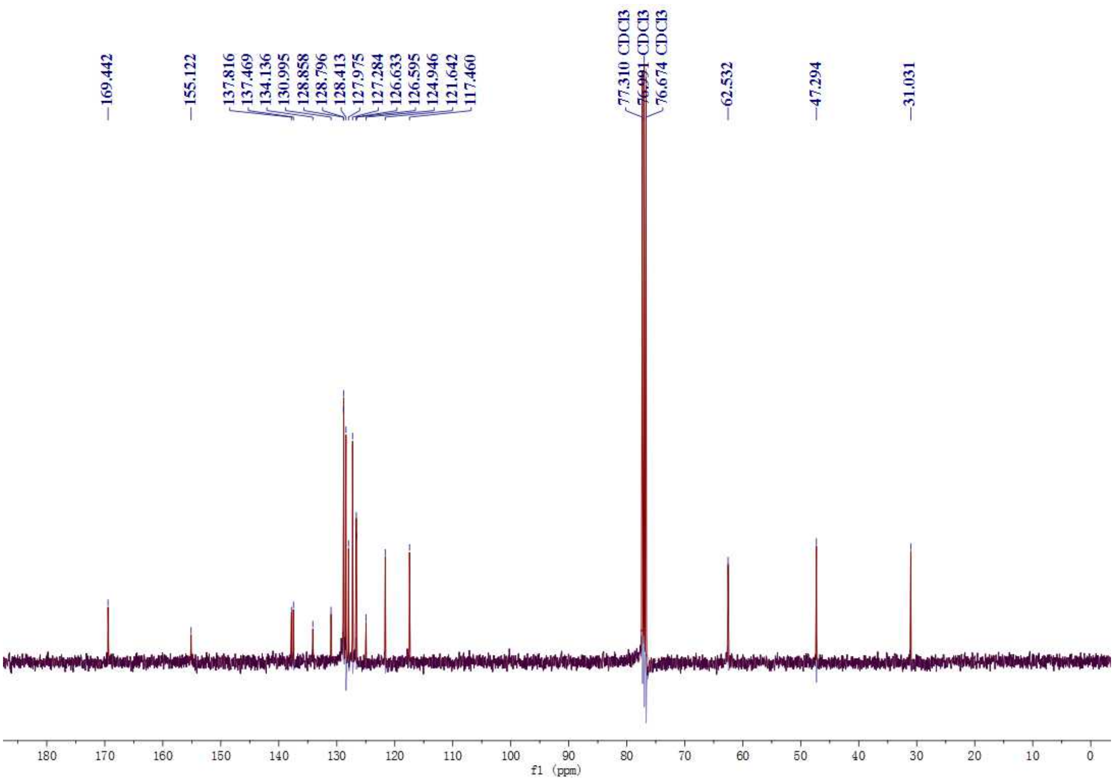

HPLC analysis: rac-**3j**

| | |
|---|---|
| Sample name: | NI 78a |
| Data file: | C:\SNOOPY\NI\78AR1IA.D |
| Description: | Laufmittel: n-Heptan/EtOH 7:3; Probe ist in LM/DCM gelöst. |
| Injection date: | 8/22/2014 2:10:35 PM |
| Acq. Analysis method: | CHIRALPAKIARNP.M |
| Column: | Chiralpak IA, (250 x 4,6) mm, 5μ, SN: IA00CE-RC036 |

Pressure at start: 30 bar Start flow: 0.500 ml/min Column oven: 29.97 °C

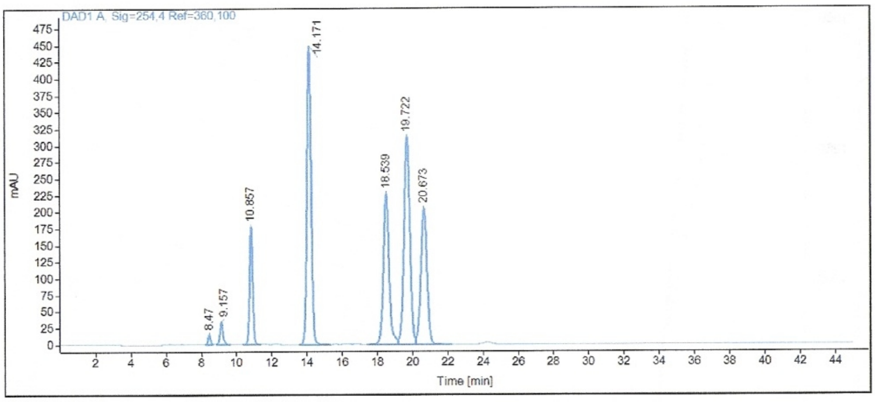


Name NI 78a

| RT [min] | Type | Area% | Area | Height | Width [min] |
|---|---|---|---|---|---|
| 8.47 | VV | 0.51 | 139.45 | 14.96 | 0.14 |
| 9.16 | VB | 1.55 | 425.39 | 34.83 | 0.18 |
| 10.86 | BV | 8.01 | 2204.42 | 178.13 | 0.19 |
| 14.17 | BB | 26.70 | 7345.37 | 448.61 | 0.25 |
| 18.54 | BV | 18.79 | 5169.10 | 226.69 | 0.35 |
| 19.72 | VV | 26.37 | 7253.87 | 312.78 | 0.36 |
| 20.67 | VB | 18.07 | 4971.92 | 204.59 | 0.38 |
| | Sum | 100.00 | 27509.52 | | |

Enantioenriched **3j**

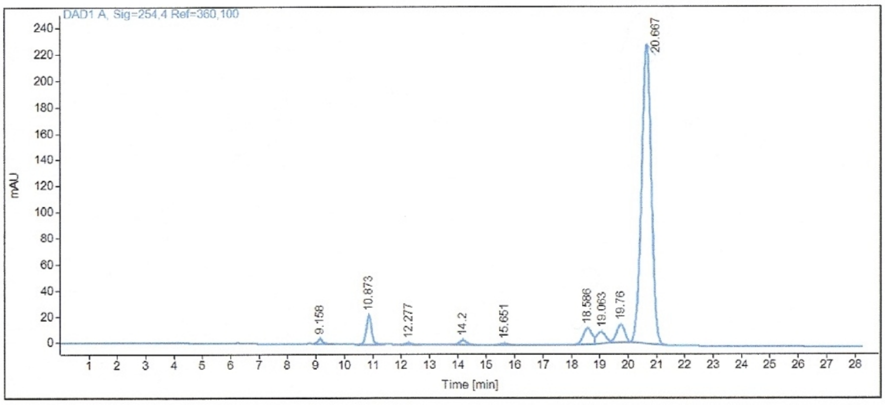


Name Ni 78 b

| RT [min] | Type | Area% | Area | Height | Width [min] |
|---|---|---|---|---|---|
| 9.16 | VV | 0.76 | 50.16 | 3.77 | 0.19 |
| 10.87 | BV | 4.29 | 283.19 | 21.98 | 0.20 |
| 12.28 | BB | 0.23 | 15.33 | 1.14 | 0.21 |
| 14.20 | BB | 0.88 | 58.26 | 3.33 | 0.27 |
| 15.65 | BB | 0.33 | 21.47 | 1.24 | 0.27 |
| 18.59 | BV | 4.01 | 264.78 | 12.11 | 0.33 |
| 19.06 | VV | 3.01 | 198.88 | 8.94 | 0.34 |
| 19.76 | VB | 4.20 | 277.37 | 13.61 | 0.32 |
| 20.67 | BB | 82.29 | 5433.83 | 228.04 | 0.37 |
| | Sum | 100.00 | 6603.27 | | |

$^{1}$H NMR of **3k**

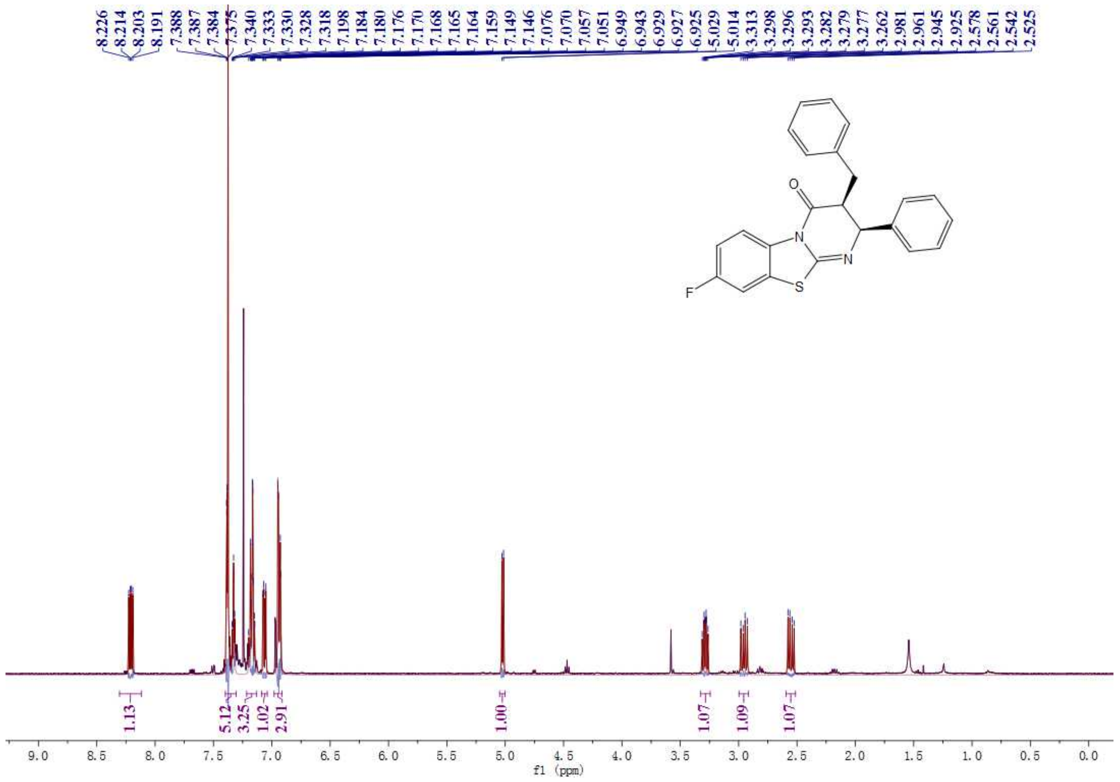

$^{13}$C NMR of **3k**

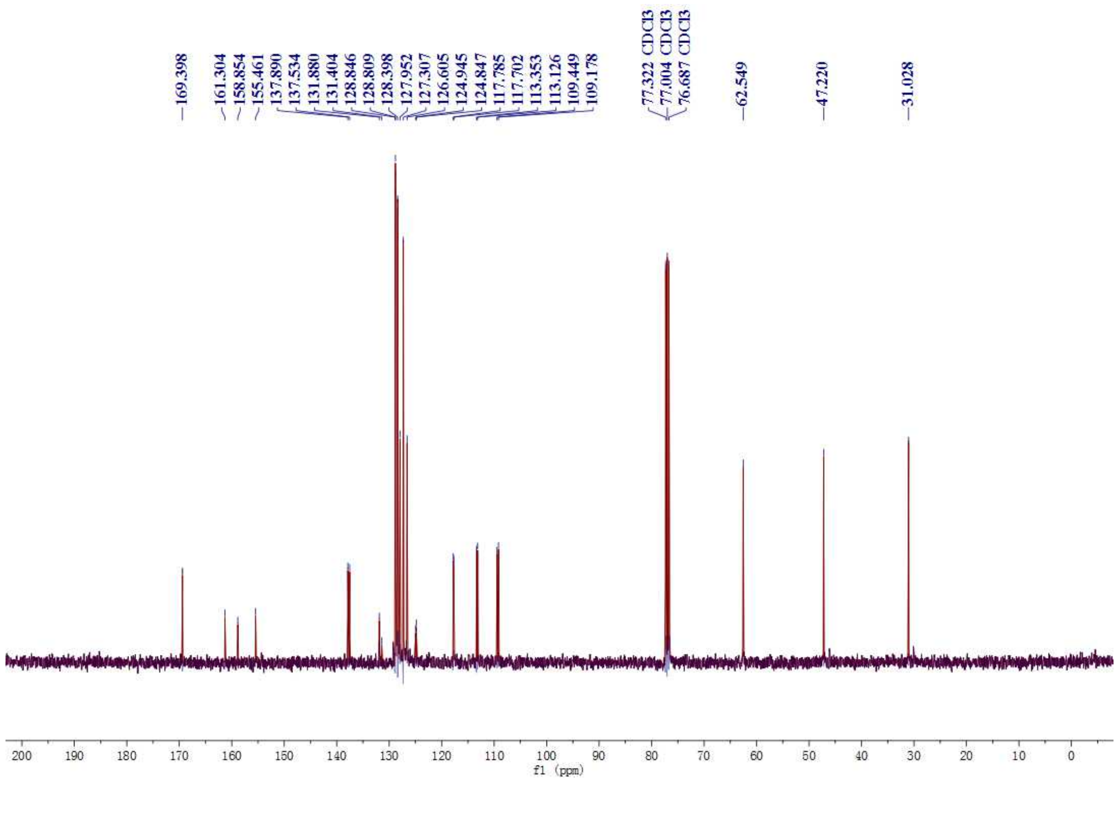

HPLC analysis: rac-**3k**

Sample name: **Ni 79 a rac**

Data file: C:\SNOOPY\NI\79AR2IA.D
Description: Laufmittel: n-Heptan/EtOH 7:3;
Probe ist in LM/DCM gelöst.

Injection date: 8/29/2014 11:32:13 AM
Acq. Analysis method: CHIRALPAKIARNP.M

Column: Chiralpak IC, (150 x 4,6) mm, 5μ, SN: IC00CD-QF015

Pressure at start: 31 bar Start flow: 0.500 ml/min Column oven: 29.99 °C

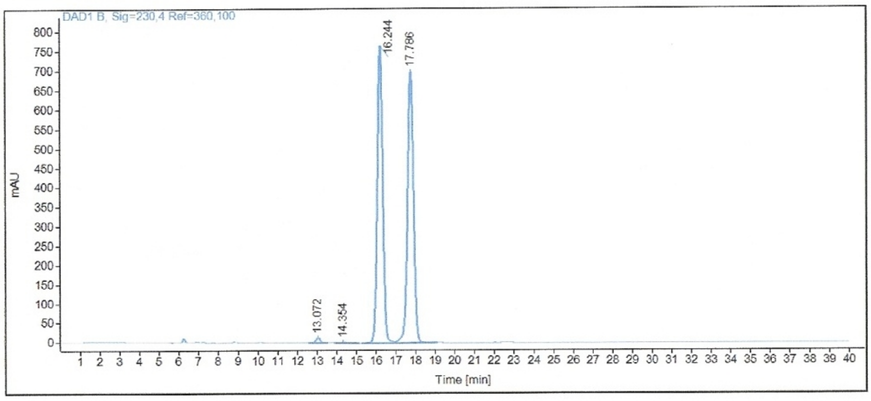


Name Ni 79 a rac

| RT [min] | Type | Area% | Area | Height | Width [min] |
|---|---|---|---|---|---|
| 13.07 | BB | 0.70 | 203.93 | 13.66 | 0.23 |
| 14.35 | BB | 0.17 | 49.85 | 2.78 | 0.27 |
| 16.24 | BV | 49.31 | 14467.52 | 764.91 | 0.29 |
| 17.79 | VB | 49.82 | 14618.54 | 699.14 | 0.32 |
| | Sum | 100.00 | 29339.84 | | |

Enantioenriched **3k**

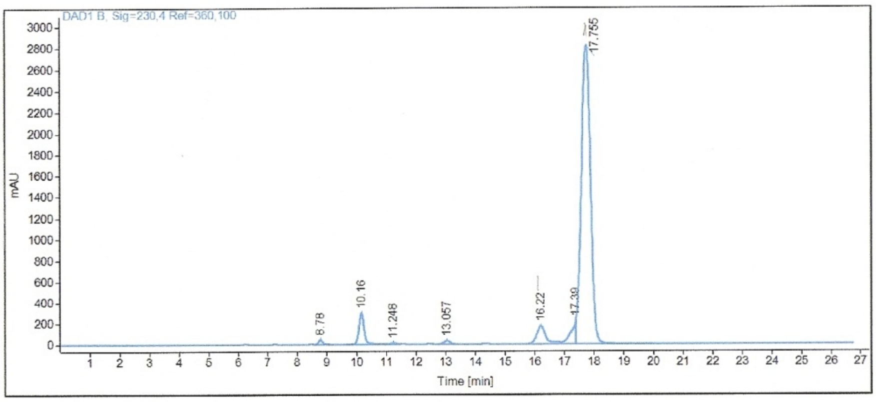


Name Ni 79 b

| RT [min] | Type | Area% | Area | Height | Width [min] |
|---|---|---|---|---|---|
| 8.78 | VB | 0.66 | 481.60 | 44.07 | 0.16 |
| 10.16 | BV | 5.08 | 3728.16 | 296.39 | 0.19 |
| 11.25 | VV | 0.29 | 213.46 | 14.60 | 0.22 |
| 13.06 | VB | 0.65 | 475.79 | 31.37 | 0.23 |
| 16.22 | VV | 5.01 | 3678.52 | 169.07 | 0.33 |
| 17.39 | MF | 3.41 | 2504.04 | 216.62 | 0.19 |
| 17.75 | FM | 84.91 | 62351.34 | 2818.94 | 0.37 |
| | Sum | 100.00 | 73432.91 | | |

$^{1}H$ NMR of **3l**

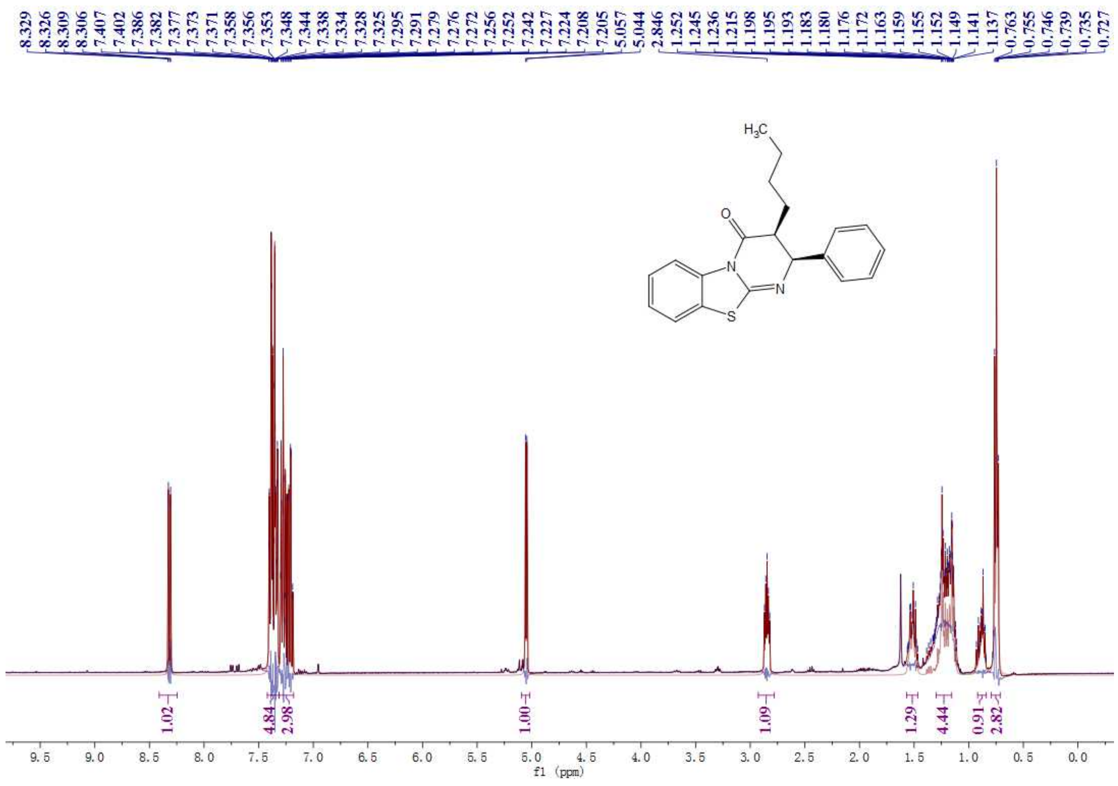


$^{13}C$ NMR of **3l**

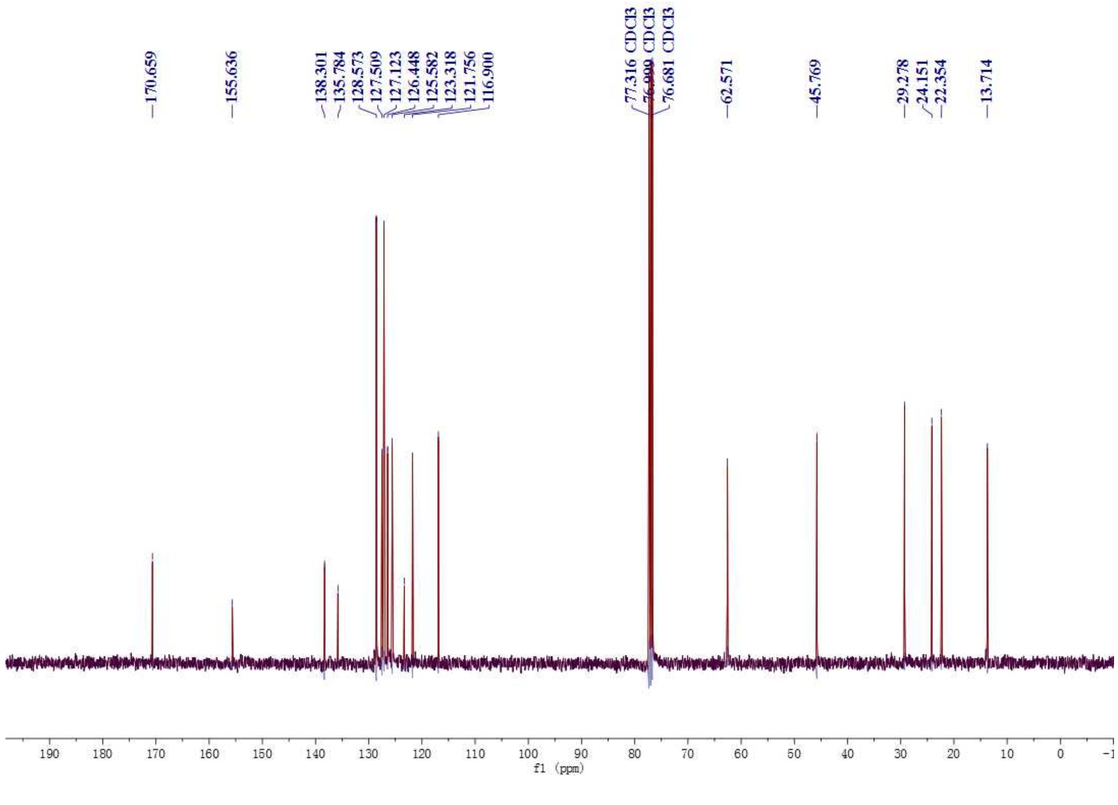

HPLC analysis: scalemic mixture of **3l** and *ent*-**3l**

**Sample name:** Ni 86 b 1+2

**Data file:** C:\SNOOPY\NI\NI 86 B 1+2 IC.D
**Description:** Laufmittel: n-Heptan/IP 9:1 Die Probe ist DCM/LM gelöst.

**Injection date:** 10/1/2014 11:43:41 AM
**Acq. Analysis method:** CHIRALPAKIC1-6LNP.M

**Column:** Chiralpak IC, (150 x 4,6) mm, 5µ, SN: IC00CD-QF015

**Pressure at start:** 23 bar **Start flow:** 0.700 ml/min **Column oven:** 30.01 °C

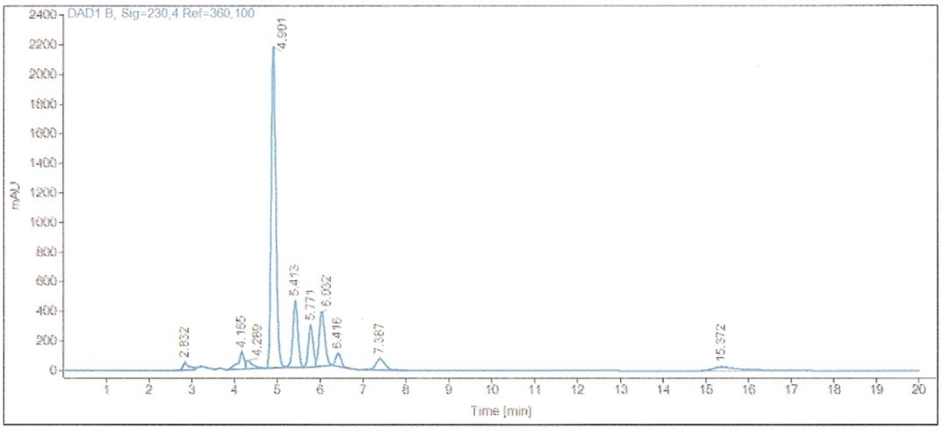


**Name** Ni 86 b 1+2

| RT [min] | Type | Area% | Area | Height | Width [min] |
|---|---|---|---|---|---|
| 2.83 | BV | 1.89 | 592.49 | 50.40 | 0.16 |
| 4.16 | BV | 3.56 | 1119.69 | 113.78 | 0.14 |
| 4.29 | VB | 1.91 | 600.60 | 54.86 | 0.16 |
| 4.90 | BV | 51.82 | 16286.05 | 2171.36 | 0.12 |
| 5.41 | VB | 11.84 | 3722.13 | 448.95 | 0.13 |
| 5.77 | BV | 7.23 | 2271.81 | 278.24 | 0.13 |
| 6.03 | VB | 10.91 | 3427.03 | 369.51 | 0.14 |
| 6.42 | BB | 2.52 | 792.90 | 88.40 | 0.14 |
| 7.39 | BB | 3.65 | 1146.49 | 73.78 | 0.24 |
| 15.37 | BBA | 4.67 | 1466.05 | 25.37 | 0.79 |
| | Sum | 100.00 | 31425.24 | | |

Enantioenriched **3l**

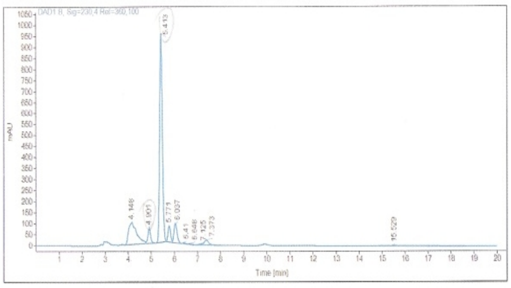


**Name** Ni 86 - B1

| RT [min] | Type | Area% | Area | Height | Width [min] |
|---|---|---|---|---|---|
| 4.15 | BV | 19.44 | 2455.99 | 99.08 | 0.31 |
| 4.90 | VB | 4.40 | 555.98 | 69.17 | 0.12 |
| 5.41 | BB | 60.75 | 7676.68 | 947.68 | 0.12 |
| 5.77 | BV | 4.55 | 575.17 | 74.37 | 0.12 |
| 6.04 | VB | 6.59 | 832.87 | 88.92 | 0.15 |
| 6.41 | BV | 0.29 | 36.80 | 4.96 | 0.12 |
| 6.65 | VB | 0.21 | 25.96 | 3.21 | 0.13 |
| 7.12 | BV | 0.30 | 37.67 | 4.62 | 0.13 |
| 7.37 | VB | 2.67 | 336.87 | 23.66 | 0.21 |
| 15.53 | BBA | 0.81 | 102.35 | 1.72 | 0.83 |
| | Sum | 100.00 | 12636.35 | | |

*ent*-**3l**

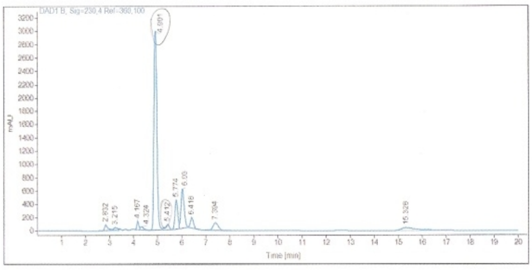


**Name** Ni 86 - B2

| RT [min] | Type | Area% | Area | Height | Width [min] |
|---|---|---|---|---|---|
| 2.83 | BV | 1.99 | 866.73 | 84.63 | 0.15 |
| 3.21 | VV | 1.14 | 496.33 | 37.24 | 0.18 |
| 4.17 | BV | 1.63 | 708.89 | 126.47 | 0.09 |
| 4.32 | VB | 0.97 | 423.66 | 42.48 | 0.15 |
| 4.90 | BV | 58.49 | 25433.26 | 2986.06 | 0.14 |
| 5.41 | VB | 2.12 | 922.02 | 78.89 | 0.17 |
| 5.77 | BV | 8.33 | 3622.00 | 436.65 | 0.13 |
| 6.03 | VB | 12.59 | 5475.42 | 590.53 | 0.14 |
| 6.42 | BB | 3.18 | 1381.26 | 152.63 | 0.14 |
| 7.39 | BB | 4.01 | 1743.86 | 112.29 | 0.24 |
| 15.33 | BBA | 5.53 | 2406.50 | 43.98 | 0.75 |
| | Sum | 100.00 | 43479.93 | | |

<sup></sup>

$^{1}$H NMR of **3m**

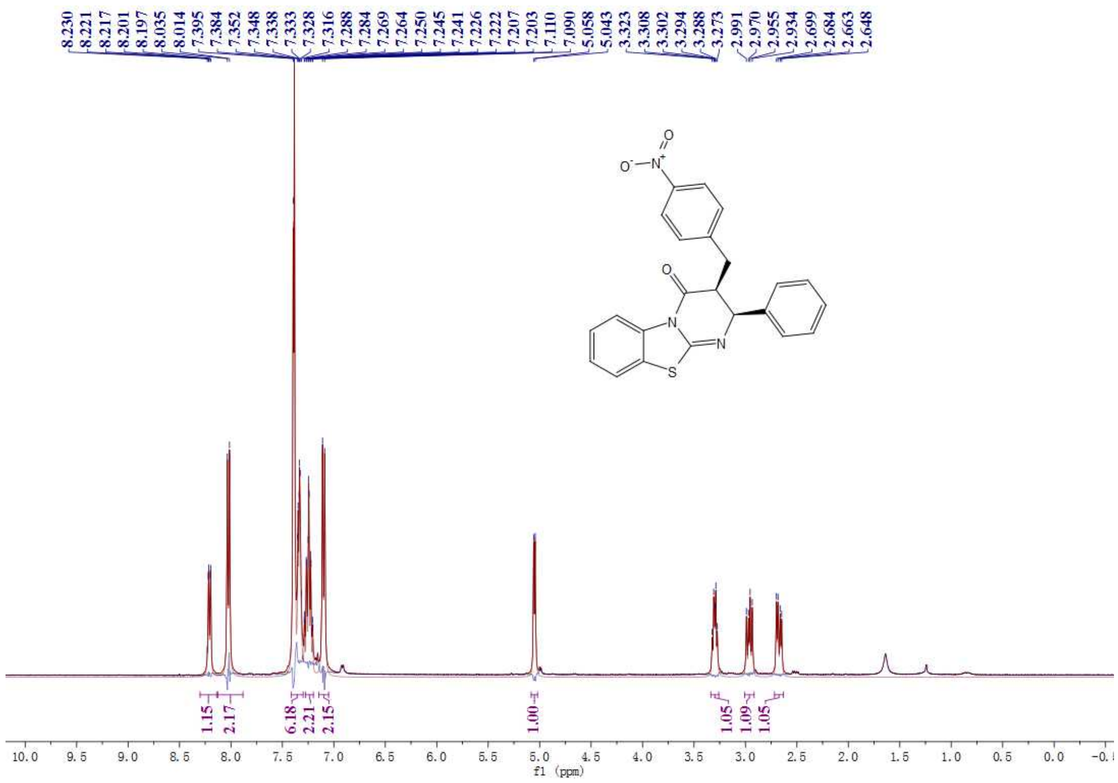

$^{13}$C NMR of **3m**

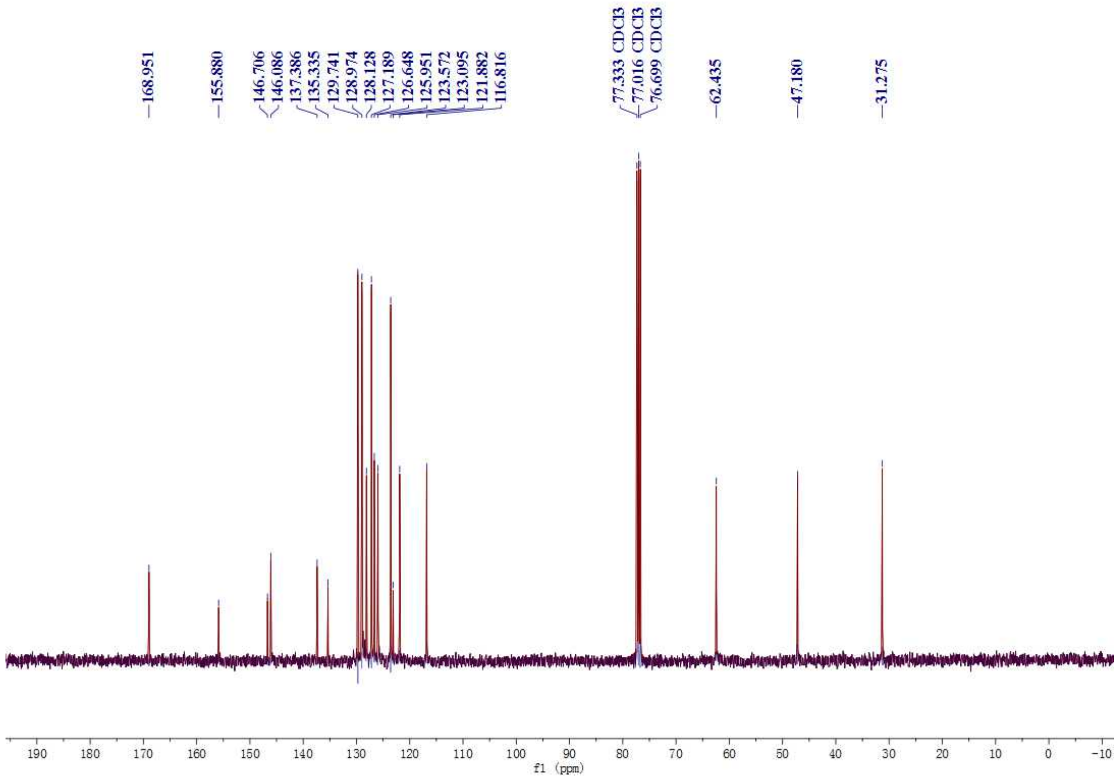

HPLC analysis: rac-**3m**

Sample name: **Ni 87 a rac**

Data file: C:\SNOOPY\NI\NI 87 A RAC IA.D

Description: Laufmittel: n-Heptan/EtOH 7:3 Die Probe ist DCM/LM gelöst.

Injection date: 9/1/2014 8:41:15 AM

Acq. Analysis method: CHIRALPAKIARNP.M

Column: Chiralpak IA, (250 x 4,6) mm, 5μ, SN: IA00CE-RC036

Pressure at start: 43 bar Start flow: 0.700 ml/min Column oven: 29.99 °C

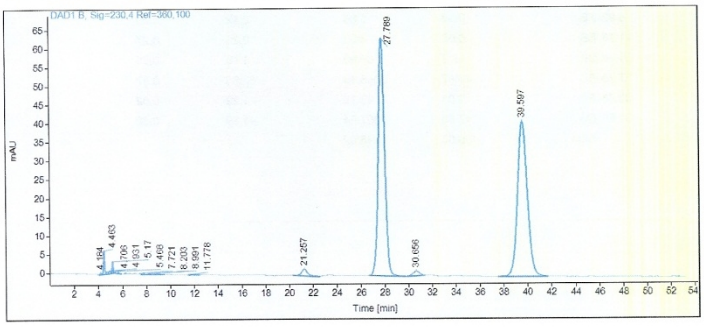

Name Ni 87 a rac

| RT [min] | Type | Area% | Area | Height | Width [min] |
|---|---|---|---|---|---|
| 4.18 | BV | 0.09 | 4.52 | 0.57 | 0.11 |
| 4.46 | VV | 0.88 | 43.69 | 6.36 | 0.10 |
| 4.71 | VV | 0.14 | 7.00 | 0.68 | 0.16 |
| 4.93 | VV | 0.14 | 6.95 | 0.79 | 0.13 |
| 5.17 | VB | 0.38 | 18.96 | 3.12 | 0.09 |
| 5.47 | BB | 0.20 | 9.94 | 0.60 | 0.22 |
| 7.72 | VB | 0.06 | 3.20 | 0.32 | 0.15 |
| 8.20 | BB | 0.04 | 2.09 | 0.21 | 0.15 |
| 8.99 | BB | 0.12 | 5.86 | 0.42 | 0.20 |
| 11.78 | BB | 0.08 | 4.03 | 0.20 | 0.27 |
| 21.26 | BB | 1.33 | 65.99 | 1.75 | 0.55 |
| 27.79 | BB | 47.87 | 2368.46 | 63.65 | 0.57 |
| 30.66 | BB | 1.01 | 49.79 | 1.22 | 0.62 |
| 39.60 | BB | 47.65 | 2357.54 | 41.39 | 0.88 |
| | Sum | 100.00 | 4948.02 | | |

Enantioenriched **3m**

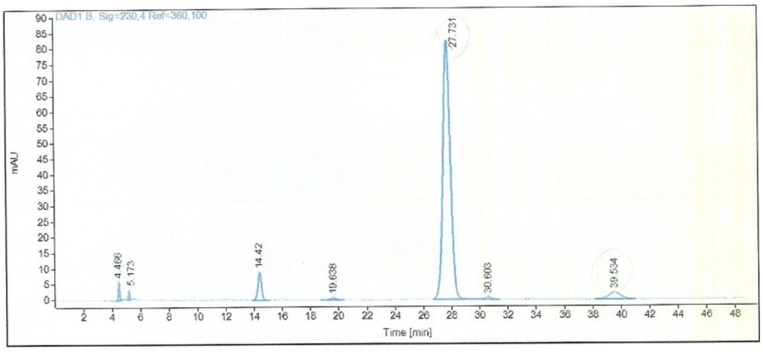

Name Ni 87 b

| RT [min] | Type | Area% | Area | Height | Width [min] |
|---|---|---|---|---|---|
| 4.47 | VV | 1.16 | 39.88 | 6.06 | 0.10 |
| 5.17 | VB | 0.55 | 19.09 | 3.22 | 0.09 |
| 14.42 | BB | 4.68 | 161.22 | 8.85 | 0.28 |
| 19.64 | BB | 0.59 | 20.25 | 0.64 | 0.45 |
| 27.73 | BB | 88.40 | 3043.43 | 82.43 | 0.57 |
| 30.60 | BB | 0.76 | 26.07 | 0.63 | 0.60 |
| 39.53 | BB | 3.86 | 132.91 | 2.20 | 0.86 |
| | Sum | 100.00 | 3442.85 | | |

$^{1}$H NMR of **3n**

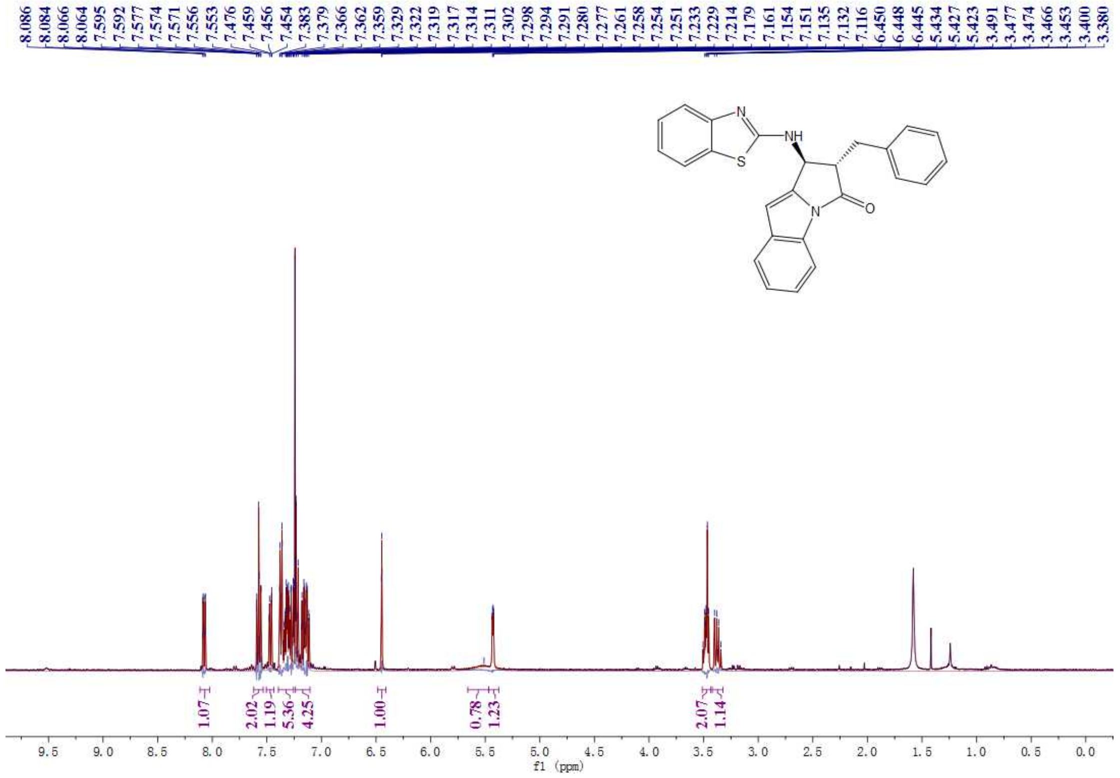


$^{13}$C NMR of **3n**

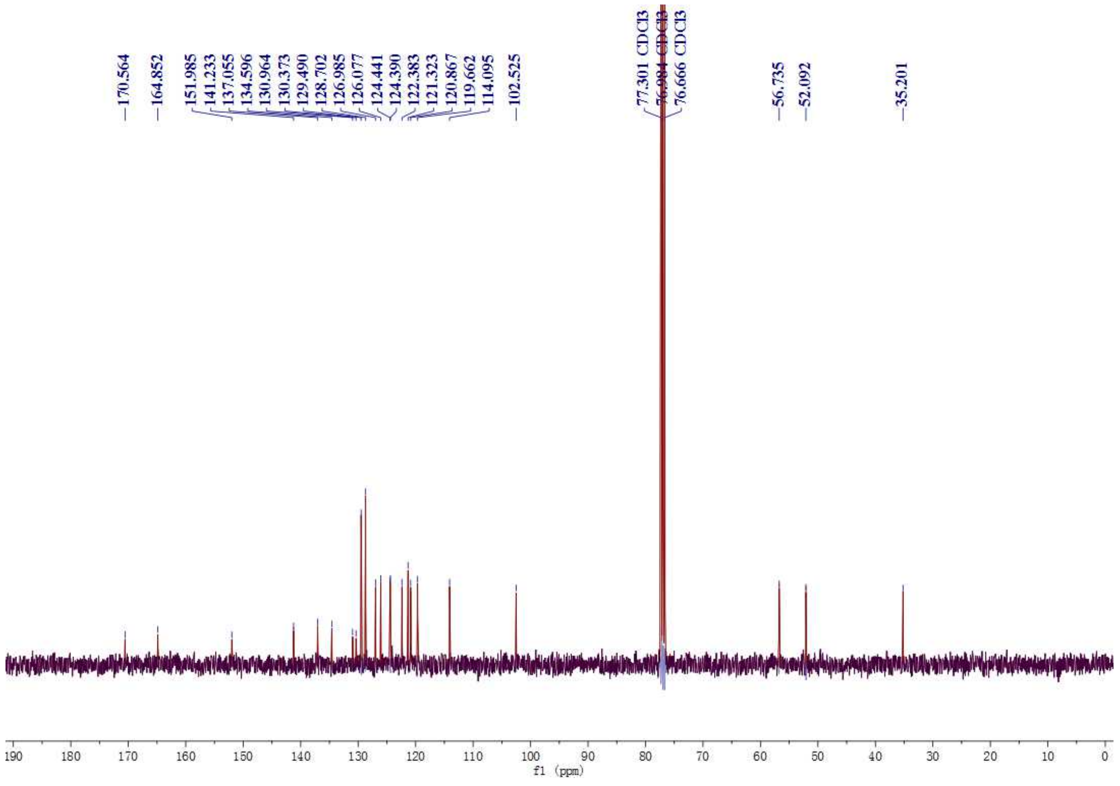

HPLC analysis: rac-**3n**

Sample name: **Ni 88 a rac**

Data file: C:\SNOOPY\NI\NI 88 A RAC IA.D

Description: Laufmittel: n-Heptan/EtOH 7:3 Die Probe ist DCM/LM gelöst.

Injection date: 9/15/2014 8:41:12 AM

Acq. Analysis method: CHIRALPAKIARNP.M

Column: Chiralpak IA, (250 x 4,6) mm, 5μ, SN: IA00CE-RC036

Pressure at start: 42 bar Start flow: 0.700 ml/min Column oven: 30 °C

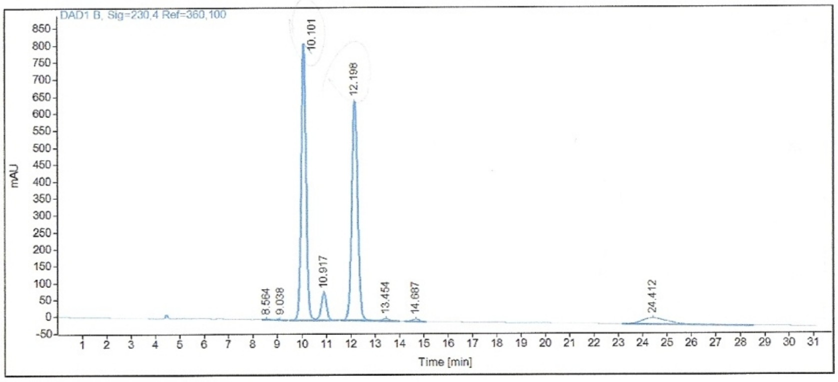


Name Ni 88 a rac

| RT [min] | Type | Area% | Area | Height | Width [min] |
|---|---|---|---|---|---|
| 8.56 | VB | 0.09 | 22.65 | 1.33 | 0.27 |
| 9.04 | BB | 0.10 | 26.14 | 1.72 | 0.23 |
| 10.10 | BV | 44.33 | 11211.09 | 816.05 | 0.21 |
| 10.92 | VB | 4.92 | 1244.05 | 81.61 | 0.23 |
| 12.20 | BV | 44.23 | 11185.47 | 649.48 | 0.26 |
| 13.45 | VB | 0.55 | 138.39 | 7.10 | 0.30 |
| 14.69 | BB | 0.49 | 123.64 | 6.15 | 0.31 |
| 24.41 | BBA | 5.30 | 1339.96 | 18.36 | 1.08 |
| | Sum | 100.00 | 25291.39 | | |

Enantioenriched **3n**

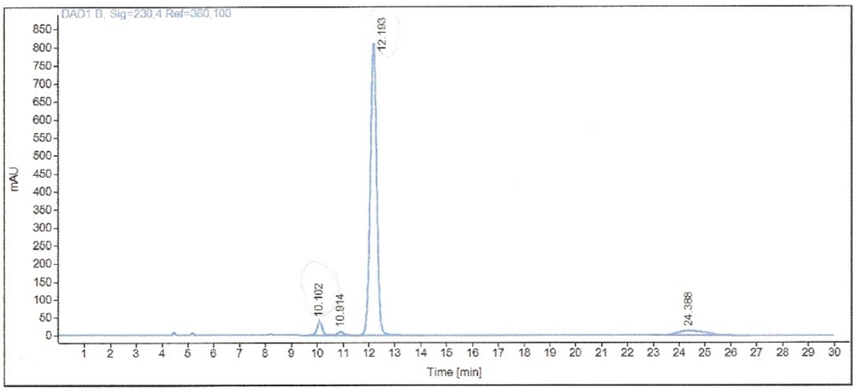


Name Ni 88 b

| RT [min] | Type | Area% | Area | Height | Width [min] |
|---|---|---|---|---|---|
| 10.10 | BV | 3.46 | 539.64 | 38.00 | 0.22 |
| 10.91 | VV | 0.92 | 143.88 | 9.17 | 0.24 |
| 12.19 | VB | 89.22 | 13913.40 | 810.27 | 0.26 |
| 24.39 | BB | 6.40 | 997.83 | 12.96 | 1.11 |
| | Sum | 100.00 | 15594.75 | | |
